# Overcoming PD-1 Inhibitor Resistance with a Monoclonal Antibody to Secreted Frizzled-Related Protein 2 in Metastatic Osteosarcoma

**DOI:** 10.3390/cancers13112696

**Published:** 2021-05-30

**Authors:** Patrick Nasarre, Denise I. Garcia, Julie B. Siegel, Ingrid V. Bonilla, Rupak Mukherjee, Eleanor Hilliard, Paramita Chakraborty, Cécile Nasarre, Jason T. Yustein, Margaret Lang, Aneese A. Jaffa, Shikhar Mehrotra, Nancy Klauber-DeMore

**Affiliations:** 1 Department of Surgery, Medical University of South Carolina, Charleston, SC 29425, USA; nasarre@musc.edu (P.N.); garciad@musc.edu (D.I.G.); siegelju@musc.edu (J.B.S.); bonillai@musc.edu (I.V.B.); mukherr@musc.edu (R.M.); hilliael@musc.edu (E.H.); chakrabp@musc.edu (P.C.); mglang@email.sc.edu (M.L.); aaj39@mail.aub.edu (A.A.J.); mehrotr@musc.edu (S.M.); 2 Department of Medicine, Medical University of South Carolina, Charleston, SC 29425, USA; nasarrec@musc.edu; 3 Department of Pediatrics, The Faris D. Virani Ewing Sarcoma Center at the Texas Children’s Cancer and Hematology Centers, Baylor College of Medicine, Houston, TX 77030, USA; yustein@bcm.edu

**Keywords:** SFRP2, CD38, WNT, NFAT, immunotherapy

## Abstract

**Simple Summary:**

Osteosarcoma (OS) is the most common bone tumor in the pediatric population, and long-term survival occurs in less than a third of the population with metastatic or recurrent tumors. Secreted frizzled-related protein 2 (SFRP2) promotes metastatic OS cell migration and tumor angiogenesis. This study aimed to assess the role of antagonizing SFRP2 with a humanized monoclonal antibody to SFRP2 (hSFRP2 mAb) in OS metastases in vivo and the role of SFRP2 in T-cells. Our results demonstrate that hSFRP2 mAb treatment inhibits metastases in two metastatic models of OS and can overcome resistance to a PD-1 monoclonal antibody. hSFRP2 mAb treatment restores T-cell proliferation and, in T-cells, inhibits NFATc3, CD38 and PD-1 expression. We conclude that SFRP2-targeted immunotherapy reduces the growth of metastatic osteosarcoma, not only through a direct antitumor and antiangiogenic effect but also by impacting the immune system.

**Abstract:**

Secreted frizzled-related protein 2 (SFRP2) promotes the migration/invasion of metastatic osteosarcoma (OS) cells and tube formation by endothelial cells. However, its function on T-cells is unknown. We hypothesized that blocking SFRP2 with a humanized monoclonal antibody (hSFRP2 mAb) can restore immunity by reducing CD38 and PD-1 levels, ultimately overcoming resistance to PD-1 inhibitors. Treating two metastatic murine OS cell lines in vivo, RF420 and RF577, with hSFRP2 mAb alone led to a significant reduction in the number of lung metastases, compared to IgG1 control treatment. While PD-1 mAb alone had minimal effect, hSFRP2 mAb combination with PD-1 mAb had an additive antimetastatic effect. This effect was accompanied by lower SFRP2 levels in serum, lower CD38 levels in tumor-infiltrating lymphocytes and T-cells, and lower PD-1 levels in T-cells. In vitro data confirmed that SFRP2 promotes NFATc3, CD38 and PD-1 expression in T-cells, while hSFRP2 mAb treatment counteracts these effects and increases NAD^+^ levels. hSFRP2 mAb treatment further rescued the suppression of T-cell proliferation by tumor cells in a co-culture model. Finally, hSFRP2 mAb induced apoptosis in RF420 and RF577 OS cells but not in T-cells. Thus, hSFRP2 mAb therapy could potentially overcome PD-1 inhibitor resistance in metastatic osteosarcoma.

## 1. Introduction

Osteosarcoma (OS) is the most common malignant bone tumor in children and adolescents [1]. If feasible, the primary tumor is resected surgically, with both neoadjuvant chemotherapy and adjuvant chemotherapy delivered. However even with standard chemotherapy, only two-thirds of patients with initially resectable disease are cured, with long-term survival occurring in <30% of patients with metastatic or recurrent tumors. The lung is involved in 80% of cases with metastatic disease and subsequent respiratory distress is responsible for most of the fatalities [2]. All patients, even nonmetastatic ones, receive high-dose cytotoxic chemotherapy treatments containing doxorubicin, cisplatin and methotrexate [3]. Unfortunately, these treatments are often toxic and not effective in eradicating disseminated metastases. Despite numerous trials with various chemotherapy and targeted therapy regimens, survival rates have been unchanged over the past 20 years [4], and therefore, there is a critical unmet need for novel therapies to improve survival for patients with metastatic osteosarcoma.

A new potential therapeutic target, secreted frizzled-related protein 2 (SFRP2), has recently been reported for metastatic osteosarcoma. High expression of SFRP2 in OS patient samples correlates with poor survival, and SFRP2 overexpression suppresses normal osteoblast differentiation, promotes OS features and facilitates angiogenesis [5]. Functional studies revealed that stable overexpression of SFRP2 within localized human and mouse OS cells significantly increased cell migration and invasive ability in vitro and enhanced metastatic potential in vivo [1]. Additionally, knocking down SFRP2 within metastatic OS cells showed a decrease in cell migration and invasion ability in vitro, therefore corroborating a critical biological phenotype carried out by SFRP2 [1].

Secreted frizzled-related protein 2 is a secreted protein involved in WNT signaling in tumor and endothelial cells. In endothelial cells, SFRP2 activates the non-canonical Wnt/Ca^2+^ pathway to stimulate angiogenesis [6,7]. The Wnt/Ca^2+^ pathway is a β-catenin-independent pathway mediated through activated G proteins and phospholipases. When this pathway is activated, it leads to transient increases in cytoplasmic free calcium and the activation of the phosphatase calcineurin that dephosphorylates the nuclear factor of activated T-cells (NFAT), which then translocate from the cytoplasm to the nucleus. NFAT mediates tumor growth, including cell growth, survival, invasion and angiogenesis [8]. NFAT proteins are not only important in angiogenesis but also have crucial roles in the development and function of the immune system and regulate T-cell activation [9]. Since NFAT has been reported to regulate T-cell activity, we hypothesized that SFRP2 could play a role in T-cell regulation and may be a therapeutic target for immunotherapy.

We recently reported the development of a novel humanized antibody to SFRP2 (hSFRP2 mAb) that was efficacious at inhibiting human triple-negative metaplastic breast cancer and murine angiosarcoma tumors in mice. Immunogenicity testing of hSFRP2 mAb in a time course T-cell study did not induce proliferative responses in any of the healthy donors. The hSFRP2 mAb bound recombinant SFRP2 with an EC_50_ of 8.7 nM and a Kd of 74.1 pM. The maximal efficacious antitumor dose in vivo was 4 mg/kg iv q 3 days, and there was no toxicity seen at 20 mg/kg iv q 3 days over 21 days of treatment. The PK studies showed a half-life of 4.1 days. Importantly, hSFRP2 mAb treatment was not associated with any weight loss, lethargy or histologic changes in the liver or kidney [10]. In the present study, we test the efficacy of this novel hSFRP2 mAb on metastatic osteosarcoma and explore a new mechanism of action examining the effects on the immune system.

Other novel therapies that have been studied for osteosarcoma include immunotherapy. Expression of PD-L1 in osteosarcoma correlates with immune cell infiltration and was found to be significantly associated with poor five-year-event-free survival [11]. Despite these findings, there was a lack of efficacy in osteosarcoma in a phase II trial of pembrolizumab (SARC028), wherein only 5% of patients with metastatic osteosarcoma had an objective response [12]. One recently reported mechanism of resistance to PD-1 antibodies in other tumor types is through the upregulation of CD38. As an ectozyme, CD38 converts NAD^+^ to ADP-ribose (ADPR) and cADPR, which are essential for the regulation of extracellular metabolites, intracellular Ca^2+^, cell adhesion and signal transduction pathways. CD38 is a major mechanism of acquired resistance to the PD-1/PD-L1 blockade, causing CD8^+^ T-cell suppression [13]. Thus, lowering CD38 expression may rescue T-cells from tumor-induced dysfunction. Since calcium and NFAT signaling regulate CD38 expression in various cell types [14], we determined whether SFRP2 inhibition would lead to a decrease in Ca^2+^/NFATc3 signaling in T-cells and whether targeting SFRP2 with hSFRP2 mAb would affect CD38 and overcome resistance to PD-1 inhibition in metastatic osteosarcoma.

## 2. Materials and Methods

### 2.1. Cell Culture

#### 2.1.1. Osteosarcoma Cell Lines

The murine osteosarcoma cell lines were established from RF420 and RF577 mouse osteosarcoma cell lines obtained from Dr. Jason T. Yustein (Texas Children’s Cancer and Hematology Centers, Department of Pediatrics, Baylor College of Medicine, Houston, TX, USA) and were established from murine osteosarcoma tumors generated using an osteoblast-conditional Cre-recombinase model with alterations in p53, as previously described [4]. Briefly, tumors were disassociated using the Miltenyi gentle MACS Dissociator for enzymatic digestion. After digestion, cells were placed through a 70 μm cell strainer, washed, resuspended in media and plated into a 60 mm plate. The resulting osteosarcoma cell lines RF420 and RF577 were authenticated and confirmed to be of mouse origin and tested for evidence of cross-species contamination (human, rat, Chinese hamster and African green monkey). In addition, the mouse cell lines were tested for mycoplasma prior to being used in vivo. All cells were cultured in DMEM (#30-202, ATCC Manassas, VA, USA) with 10% heat inactivated FBS (#BT 201-500-D, BioFluid, Fleming Island, FL, USA) and 1% penicillin/streptomycin (#MT30009C, Thermo Fisher Scientific, Waltham, MA, USA). All cell lines were cultured at 37 °C, 5% CO_2_ and 95% humidity. Cells were tested for rodent pathogens by Charles River Research Animal (Wilmington, MA, USA) before injection in vivo.

#### 2.1.2. Splenic T-Cell Isolation for Culture

Mouse splenic T-cells were obtained from C57BL/6 (Envigo Indianapolis, IN, USA) or PMEL mice (The Jackson laboratory, Bar Harbor, ME, USA) and euthanized following a protocol approved by the Institutional Animal Care and Use Committee (IACUC). A single T-cell suspension was made by morcellating spleens through the filter of a 70 µm cell strainer (#2236548 Thermo Fisher Scientific) and washing the filter with T-cell medium RPMI, 4 mM glutamine, (#SH30228.01, GE Healthcare-Hyclone laboratories, South Logan, UT, USA), 10% FBS, 0.01 mg/mL bovine insulin (#I0156, Sigma-Aldrich, St Louis, MO, USA), 100 IU penicillin/1000 μg/mL streptomycin (#30-001-CI, Corning, Corning, NY, USA), 55 μM beta-mercaptoethanol, (#21985-023, Thermo Fisher Scientific), 1× HEPES (#SH30237.01, GE Healthcare-Hyclone laboratories), 1× non-essential amino acids (#1681049, MP Biomedicals, Solon, OH, USA), 1× sodium pyruvate (Cat# 116-079-721, Quality Biological, Gaithersburg, MD, USA), 1× RPMI 1640 vitamins solution (#R7256, Sigma-Aldrich).

The cell suspension was then centrifuged at 500× *g* for 5 min and resuspended in PBS, centrifuged and resuspended in PBS twice again at 500 g for 5 min, and incubated for 1 min RT in 1 mL ACK lysis buffer (#118-156-101, Quality Biological). Cells were then resuspended in PBS with 1% FBS to stop the reaction, centrifuged, resuspended in T-cell medium and counted using trypan blue (#145003) on the TC-20 Cell Counter, both from Bio-Rad (Hercules, CA, USA), and placed at the desired concentration in T-cell medium supplemented with IL-2 (6000 U/mL) (NCI repository, 10^6^ units resuspended in 1 mL 0.9% NaCl). IL-2 was added to the T-cell medium throughout our experiments for the maintenance of naïve T-cells.

For the combined isolation of CD4^+^ and CD8^+^ T-cells necessary to generate the results for T-cell assays to evaluate whether the hSFRP2 mAb effects apoptosis and TGFβ-induced elevation of CD38 and PD-1 in T-cells, splenocytes were first isolated from C57BL/6 mice, resuspended in T-cell medium, and centrifuged at 500 *g* for 5 min. CD4^+^ and CD8^+^ T-cells were then isolated by negative subtraction using the following mix of biotinylated antibodies diluted at 1:200: TER119 (#116204), CD25 (#102004), GR-1 (#108404), NK1.1 (#108704), CD11C (#117304), CD11B (#101204), CD19 (#101504), all from BioLegend (San Diego, CA, USA) and incubated on ice for 15 min. Cells were then incubated for 20 min RT on a magnetic tube holder with 200 µL of a streptavidin-bound beads solution (#557812) from BD Biosciences (Franklin Lakes, NJ, USA). CD4^+^ and CD8^+^ cells were isolated from the supernatant and other cells bound to the beads were discarded. Cells were finally counted and incubated in T-cells medium + IL-2. Finally, CD4^+^ and CD8^+^ cells were specifically identified by flow cytometry using anti-CD4 FITC (1:100; #100406) and anti-CD8 APC (1:200; #100712) from BioLegend (see Section 2.10.2. for more details).

For the specific isolation of CD8^+^ T-cells necessary to generate the results for tumor-induced suppression of T-cells, the Dynabeads Untouched Mouse CD8^+^ Cells kit was used following the manufacturer’s protocol (#11417D, Invitrogen, Waltham, MA, USA).

### 2.2. Reagents

Recombinant human SFRP2 protein (SFRP2) was prepared as previously described [15] and provided by the Protein Expression and Purification Core facility from University of North Carolina, Chapel Hill. Humanized SFRP2 monoclonal antibody (hSFRP2 mAb) was produced as previously described [10] and purified to remove endotoxin. A control IgG1, omalizumab (#NDC 50242-040-62), was purchased from Novartis (Basel, Switzerland). An anti-mouse PD-1/CD279 monoclonal antibody was purchased from Bioxcell, Lebanon, NH, USA (#BE0273).

### 2.3. Western Blot Analysis

A minimum of 5 × 10^6^ osteosarcoma cells or 10^7^ splenic T-cells were used for Western blot analysis. For the analysis of endogenous proteins levels, osteosarcoma cells were processed for a Western blot without any preliminary treatment. To study the response to SFRP2 protein, naïve T-cells were maintained in T-cell medium supplemented with 6000 U/mL IL-2 and treated for 1 h with or without SFRP2 (30 nM). To study the response to hSFRP2 mAb, splenic T-cells isolated from PMEL mice were cultured with IL-2-supplemented T-cell medium, activated by gp100 for 72 h (0.87 µM), and then treated with or without hSFRP2 mAb (10 μM) for 1 h. Splenic T-cells isolated from C57BL/6 mice were cultured with IL-2-supplemented T-cell medium and were incubated overnight in TCR at 4 °C (in anti-CD3 (2 µg/mL) and anti-CD28 (5 µg/mL)-coated wells).

Cells were washed twice with PBS and centrifuged at 500× *g* for 5 min. Cell pellets were resuspended in 200–400 µL RIPA buffer (#R3792; TEKnova, Hollister, CA, USA), kept on ice for 20 min, and sonicated for 45 s. When nuclear and cytoplasmic fractions needed to be separated, cell suspension was processed using a NE-PER nuclear and cytoplasmic extraction reagent following the manufacturer protocol (#78835, Pierce Biotechnology, Rockford, IL, USA). Protein concentration was measured using the Bio-Rad Protein Assay (#500-0205, Bio-Rad Laboratories). Samples containing equal amounts of total protein were run on 10% bis-tris SDS-PAGE gels (#NW00100BOX Invitrogen) for 1 h at 140 V. Proteins were then transferred to a PVDF membrane (#LC2005 Life Technologies; Carlsbad, CA, USA) at 30 V for 1 h 10 min. Membranes were incubated for 30 min RT in blocking buffer (PBS, Twin 0.1%, 5% milk), and incubated o/n at 4 °C with the following primary antibodies diluted in blocking buffer (PBS, 1% Tween, 5% milk): rabbit anti-CD38 (1:1000; #14637s), rabbit anti-histone H3 antibodies (1:5000; #2650s), and β-catenin (1:1000; #9582S), all from Cell Signaling (Danvers, MA, USA); rabbit anti-FZD5 (1:1000; #H00007855-D01P, Abnova, Taipei city, Taiwan); mouse anti-PD1 (1:1000; #66220-1, Proteintech, Rosemont, IL, USA); rabbit anti-NFATc3 (1:1000; #SAB2101578) and rabbit anti-actin (1:5000; #A2103) from Sigma-Aldrich; rabbit anti-TATA antibody (1:5000; #ab63766), rabbit anti-SFRP2 (1:1000; #ab86379) and rabbit anti-GAPDH (1:5000; #ab9485) from Abcam, (Cambridge, MA, USA); and PD-L1 (#PA5-20343) from Invitrogen. The following horseradish peroxidase (HRP)-conjugated secondary antibodies were used (concentration 1:5000, 1 h incubation RT): anti-mouse (#7076) from Cell Signaling and anti-rabbit (#403005, Southern Biotech, Birmingham, AL, USA). The ECL Prime Western Blotting System was used to visualize protein bands (#RPN2232; GE Healthcare). Densitometry was finally performed on imageJ, comparing loading controls and proteins of interest. Densities were calculated by multiplying the average intensity by the surface of each band. For each marker, densities were normalized to the loading control to eliminate intersample variability using the following formula: marker’s total density ×100/loading control’s total density. Final results were obtained by normalizing each value to untreated controls.

### 2.4. Defining Mouse Model to Identify Timing of Establishment of Lung Metastases

All animal studies were reviewed and approved by the Institutional Animal Care and Use Committee at MUSC and carried out in accordance with the National Institutes of Health guide for the care and use of laboratory animals. Equal numbers of male and female mice were used in all experiments. RF420 osteosarcoma cells (5 × 10^5^/100 μL) filtered through a 40 μm cell strainer were suspended in sterile PBS and injected iv in the tail vein of 6–8-week-old C57BL/6 mice (Envigo, Indianapolis, IN, USA; *n* = 3). One and two mice were euthanized at day 5 and 7, respectively; their lungs were resected, fixed overnight in 10% formalin, transferred in 70% ethanol, embedded in paraffin and sliced in 6 µm sections. Sections were deparaffinized and stained with hematoxylin and eosin (H&E) to identify the earliest occurrence of microscopically detectable metastases. Metastases were identified by H&E on day 7, and therefore for subsequent experiments with this cell line, treatment was started on day 8.

A second metastatic osteosarcoma model was used after tail vein injection in C57BL/6 mice of 5 × 10^5^/100 µL RF577 osteosarcoma cells initially filtered through 100 μm cell strainer and resuspended in sterile HBSS solution (*n* = 4). Two mice per time point were euthanized on days 8 and 11, and lung metastases were detected by H&E staining on day 11 in both mice. Therefore, for subsequent experiments with the RF577 cell line, treatments were started 12 days after injection.

### 2.5. hSFRP2 mAb Monotherapy for Osteosarcoma Lung Metastases

RF420 OS cells (5 × 10^5^/100 μL) filtered through a 40 μm cell strainer were suspended in sterile PBS and injected in the tail vein of 6–8-week-old male and female C57BL/6 mice. Intravenous treatments of mice were initiated with either IgG1 control (4 mg/kg weekly; *n* = 7) or hSFRP2 mAb (4 mg/kg every three days; *n* = 10) 8 days after RF420 cell injection. Mice weights were recorded every 3–7 days. After 21 days of treatment, animals were euthanized, and lungs and spleens were collected. Lung surface nodules were manually counted from pictures of full lungs taken immediately after resection. This experiment was repeated a second time with the same timeline, treatments and dosing (*n* = 12 for the IgG1 control group; *n* = 12 for the hSFRP2 mAb-treated group), and the results of the two experiments were combined (*n* = 19 for IgG1 control group; *n* = 22 for the hSFRP2 mAb-treated group).

### 2.6. hSFRP2 mAb and PD-1 mAb Combined Therapy in Two Metastatic Osteosarcoma Cell Lines In Vivo

RF420 cells were filtered, resuspended at 5 × 10^5^ cells/100 μL in sterile PBS, and injected i.v. in the tail vein of 6–8-week-old C57BL/6 male and female mice. Mice were randomly distributed in 4 groups: IgG1 control (omalizumab; *n* = 13), hSFRP2 mAb (*n* = 11), mouse PD-1 mAb (mPD-1 mAb) (*n* = 12) and mPD-1mAb + hSFRP2 mAb (*n* = 12). Treatment started 8 days after tumor cell inoculation. Dosage, delivery route and frequency per mouse were as follow: IgG1 control: 4 mg/kg iv, once weekly; hSFRP2 mAb: 4 mg/kg iv, every 3 days; PD-1 mAb: 200 μg/100 μL intraperitoneal injection (i.p.), every 3 days. Mice weights were recorded every 3–7 days. After 3 weeks of treatment, animals were euthanized, and their lungs were resected. Lung surface nodules were manually counted from pictures of full lungs taken immediately after resection. Lungs were then paraffin embedded, sectioned and H&E stained. Lung metastases counts were performed manually at 20× by standard light microscopy on 3 independent slices separated from each other by an interval of 200 µm.

In the second metastatic osteosarcoma model, 5 × 10^5^ RF577 tumor cells/100 μL previously filtered and resuspended in PBS were injected in the tail vein of C57BL/6 mice. Treatments began 12 days after the injection of tumor cells in four treatment groups: IgG1 control (*n* = 13), hSFRP2 mAb (*n* = 15), mPD-1 mAb (*n* = 14) and mPD-1mAb + hSFRP2 mAb (*n* = 14). Treatment continued for 7 weeks at the same dosing and frequency as described in the RF420 model. Weights were recorded every 3–7 days. When the first control animals started to display signs of illness (labored breathing, 20% weight loss, difficult ambulation or hunched posture), which happened after 49 days of treatment, mice were euthanized, lungs and spleens were collected and photographs of the lungs were taken to count the number of surface nodules. Splenic T-cells were analyzed by Western blot and flow cytometry for PD-1 and CD38 expression as described above.

### 2.7. Immunohistochemistry

Paraffin-embedded tumors from the IgG1 control-treated (*n* = 6) and hSFRP2 mAb-treated (*n* = 6) metastatic osteosarcoma mice were sectioned at 6 µm, and immunohistochemistry was performed as described previously (34). Briefly, slides were deparaffinized and rehydrated using Discovery wash (950-510, Roche Tissue diagnostics, Indianapolis, IN, USA). Antigen retrieval was performed using EDTA buffer solution for 32 min on the Discovery Ultra staining platform. Endogenous peroxidase was blocked with Discovery Inhibitor solution (760-4840, Roche Tissue Diagnostics), and the tumors were then incubated with CD31 antibody (1:200, #ab28364, Abcam) for 1 h RT. A negative control with no primary antibody added was generated to ensure the specificity of the staining. Slides were then incubated with an HRP-conjugated OmniMap anti-rabbit secondary antibody (1:200, #760-4311, Ventana Medical System, Tucson, AZ, USA) for 20 min at 37 °C, and then precipitated with a DAB substrate following chromomap DAB kit protocol instructions (#760-159, Ventana Medical Systems). Five fields per tissue slice were analyzed from pictures taken at 40× using the EVOS FLc Digital Imaging System (Thermo Fisher Scientific), and hot spots of positively stained cells were counted within each field as previously published [16]. The numbers obtained for all 5 fields were then averaged.

### 2.8. Blood Collection and Serum Preparation for the Quantification of SFRP2 Concentration in Mouse Serum by ELISA

Blood from C57BL/6 control mice (*n* = 3) or mice bearing RF420 metastases (*n* = 3), as well as blood from non-tumor-bearing mice (*n* = 8) and RF577-bearing mice treated with IgG1 (*n* = 9), hSFRP2 mAb (*n* = 12), PD-1 mAb (*n* = 8) or a combination of both antibodies (*n* = 12) was collected from the inferior vena cava immediately after euthanasia and laparotomy. The separation of serum was done using BD Vacutainer EDTA SST tubes (#367981; Becton Dickinson and Company, Franklin Lakes, NJ, USA). Following the manufacturer’s protocol, blood was allowed to clot for 30 min, centrifuged at 1300× *g* for 15 min, and serum samples were collected. Serum samples from RF420-bearing mice were processed using the MyBioSource Mouse SFRP2 ELISA kit (#MBS162693; San Diego, CA, USA), and the RayBiotech Mouse SFRP2 ELISA kit (ELM-SFRP-2; Peachtree Corners, GA, USA) was used for the RF577-bearing mice. The manufacturers’ protocols were followed for both kits. Finally, absorbance was read at 450 nm with a Synergy 2 plate reader, using Gen5 2.06 software (BioTek Instruments, Winooski, VT, USA).

### 2.9. Isolation of Mouse Osteosarcoma Tumor-Infiltrating Lymphocytes (TILs)

Lungs with metastatic tumors were removed, cut into 2–3 mm^3^ pieces that were resuspended in RPMI, then centrifuged at 500 *g* for 5 min. Pellets were then incubated in Collagenase D (#11088858001; Sigma-Aldrich) at 37 °C and 95% humidity for 1 h on a rocker, resuspended in 15 mL PBS, and filtered with a 70 µm cell strainer (#2236548; Thermo Fisher Scientific). After centrifugation at 500 *g* for 5 min, cells were resuspended in RPMI and a solution of Ficoll-Paque (3v RPMI/1v Ficoll-Paque; #17-1440-02; GE Healthcare) was slowly deposited at the bottom of the tube. The solution was centrifuged at 1000× *g* for 20 min with no centrifuge brake and acceleration set at a minimum. The fraction containing TILs at the interface between RPMI and Ficoll was removed and resuspended in PBS with 1% FBS, centrifuged at 500 *g* for 5 min RT, and resuspended in PBS. Cells were then counted, and 5 × 10^5^ cells/condition were used for staining and flow cytometry analysis (see protocol below).

### 2.10. Flow Cytometry

#### 2.10.1. Screening of Dysfunction Markers in T-cells Isolated from Metastatic Osteosarcoma

Immediately following the euthanasia of RF420 tumor-bearing mice, splenocytes and TILs from lungs were collected fresh for T-cell isolation or flow cytometry (See TIL isolation protocols above). Then, 3 × 10^5^–7 × 10^5^ splenocytes or TILs from the IgG1 and hSFRP2 mAb-treated groups were incubated in FACS buffer (0.1% Bovine Serum Albumin (BSA) in PBS) for 30 min at 4 °C (see FACS staining protocol) with the following primary antibodies: Brilliant violet 711 anti-CD103 (1:200, clone 2E7, #121435), PerCP anti-CD5 (1:200, clone 53-7.3, #100624), PE anti-CD38 (1:200, #102707), PE anti-PD1 (1:200; #109103), all from Biolegend, and PE/Cy5 anti-CD38 (1:200, #15-0381-81) from Invitrogen (Carlsbad, CA, USA). After 20 min of incubation RT, cells were centrifuged at 500 *g* for 5 min and finally fixed in 4% paraformaldehyde for 10–15 min before being resuspended in 250 µL of FACS buffer. CD8-gated T-cells were screened for mean fluorescence intensity (MFI) levels on LSRFortessa and analyzed with FlowJo v10 software (Tree Star, OR, USA).

#### 2.10.2. Staining of Extracellular Markers

From a single cell suspension, 3 × 10^5^–7 × 10^5^ T-cells were resuspended in PBS and incubated in live/dead solutions following the Live/Dead Fixable Dead Cell Stain Kit instructions (#L34963, Invitrogen). Cells were then washed with PBS, centrifuged at 500 *g* for 5 min and resuspended in a master mix of antibody and 0.2% BSA staining buffer (50 µL/sample) containing anti-CD38 PE/Cy5 (1:200; #15-0381-81, Invitrogen), anti-CD4 FITC (1:100; #100406), anti-CD8 APC (1:200; #100712) and anti-PD1 PE (1:200; #109103), all from BioLegend. After 20 min of incubation RT, cells were centrifuged at 500 *g* for 5 min and finally fixed in 4% paraformaldehyde for 10–15 min before being resuspended in 250 µL of staining buffer.

#### 2.10.3. Staining of Intracellular Markers

Any intracellular staining was performed immediately following any extracellular staining by first washing the cells in FACS buffer (PBS, 2% FBS, 10% sodium azide), resuspending them in 100 µL BD cytofix/cytoperm buffer (#5523, FOXp3/transcription factor staining buffer, Invitrogen) and incubating them at 4 °C for 20 min. Then, 100 µL of 1× BD permeabilization buffer (#8333-56 eBioscience, San Diego, CA, USA) was added directly to the sample, and cells were centrifuged at 1500 RPM and at 4 °C for 5 min. Cells were resuspended in permeabilization buffer and centrifuged before incubating with the PE/Cyanine 7 TNFα (#506323 clone MP6-XT22, BioLegend, San Diego, CA, USA) for 30 min. Following this incubation, 150 µL of permeabilization buffer was added, and cells were centrifuged at 500 *g* for 5 min, then washed with PBS and resuspended in 200 µL of FACS buffer. An unstained control, containing just cytofix/cytoperm buffer and permeabilization buffers with no antibody, was run for each condition.

#### 2.10.4. Compensation

For each type of staining, compensations were performed on ArC reactive or negative beads (#A10628) for live/dead staining or with OneComp eBeads (#1923777), all from Invitrogen. Staining was analyzed by flow cytometry using the BD LSRFortessa system (BD Biosciences, Franklin Lakes, NJ, USA). Ten thousand reads/sample were captured, and positively stained cells were counted by FACS on the BD LSRFortessa platform. Analysis was done using FlowJo v10 software (Tree Star, OR, USA).

### 2.11. Real Time RT-PCR

Splenic T-cells, in the amount of 6 × 10^6^, in single cell suspension isolated from C57BL/6 mice were incubated in complete T-cell medium with 6000 U/mL IL-2 and 10% FBS overnight and with or without SFRP2 (30 nM) for 1 h. T-cells were then centrifuged at 500 *g* for 5 min and washed twice with PBS. RNA was purified using RNeasy Plus Micro Kit (#74034, Invitrogen, Carlsbad, CA, USA). Superscript III First-strand SuperMix cDNA synthesis (#11752-050, Invitrogen) and Fast SYBR Green qRT-PCR (#4385612, ABI, Bedford, MA, USA) were performed as recommended by the manufacturers. Primer sequences were the following: CD38 forward: 5′-AGCCTGTGTTGTCGTCTAGC-3′, CD38 reverse: 5′-ACACCTATTCCAGCAAGGCC-3′; PD-1 forward: 5′-GCAATCAGGGTGGCTTCTAGA-3′, PD-1 reverse: 5′-TTGGACAAGCTGCAGGTGAA-3′. Results are reported as fold change compared to the control. Each sample in each qRT-PCR was triplicated. Three independent experiments were performed, and each of the three sets of samples was retested 3 times for a total n of 9.

### 2.12. Treatment of TGF-β-Induced CD38^Hi^ T-Cells with hSFRP2 mAb

Splenic T-cells were plated in TCR (anti-CD3 (2 µg/mL) and anti-CD28 (5 µg/mL)) pre-coated plates. All of the following conditions were done in triplicate and in culture with 6000U/mL IL-2 for 72 h. Negative controls contained only T-cells in IL-2-enriched media. In the remaining conditions, in addition to IL-2, each experimental well with T-cells contained the following experimental conditions: TCR alone, TCR with or without TGF-β (5 ng/mL), TCR with or without hSFRP2 mAb (10 μM). The final condition included TCR stimulated cells with both TGF-β (5 ng/mL) and hSFRP2 mAb (10 μM). Conditions with TGF-β were incubated with it for the full 72 h of culture. For any condition with both hSFRP2 mAb and TGF-β, hSFRP2 was added either 1 h after TGF-β and kept in for the duration of the experiment or hSFRP2 mAb was added 24 h after TGF-β. Following the experiment, cells were counted and either stained for FACS analysis and analyzed by flow cytometry, lysed for Western blot or processed for NAD analysis.

### 2.13. NAD^+^ Quantitative Assay

For NAD^+^ analysis, at least 2.5 × 10^5^ T-cells were required and processed immediately following the NAD^+^ protocol following the manufacturer’s protocol of the NAD/NADH cell-based assay kit (#600480, Cayman Chemical, Ann Arbor, MI, USA). Briefly, cells were centrifuged at 500 *g* for 5 min and then incubated under agitation with a permeabilization buffer for 30 min. After centrifugation at 1000× *g* for 10 min, samples were transferred onto a 96-well plate and 100 µL standards (purified NAD^+^) were added in separate wells. Samples and standards were then incubated with a reaction buffer for 1 h 30 min RT under agitation. Optical densities were finally read at 450 nm using the Synergy 2 plate reader and using Gen5 2.06 software (BioTek Instruments, Winooski, VT, USA).

### 2.14. Effects of hSFRP2 mAb on Apoptosis in Tumor Cells and T-Cells

OS cell lines (RF420 and RF577) and T-cells were plated in 96-well plates (#0030730119; Eppendorf, Hamburg, Germany) at 1.5 × 10^4^ and 1.0 × 10^4^ cells/well, respectively. The next day, cells were treated for 1 h with 10 μM of hSFRP2 mAb or 10 μM of IgG1 (Omalizumab) control at 37 °C and 5% CO_2_. Apoptosis was measured following the protocol of the Apoptotic Detection kit (#PK-CA707-30017; PromoCell, GmbH, Heidelberg, Germany). Apoptotic cells were positive for FITC, and necrotic cells were positive for Texas Red. Images were acquired using the 10× objective lens of the EVOS FLc Digital Imaging System (Thermo Fisher Scientific). Cells were counted using ImageJ cell counting software. Each data point was the result of 3 independent experiments, each containing 4 separate wells (*n* = 12).

### 2.15. Proliferation of T-Cells in Co-Culture with Osteosarcoma Cells

RF420 cells were plated at 3000 cells/well and incubated with either IgG1 control (10 µM) or hSFRP2 mAb (10 µM) for 72 h (*n* = 6). The number of healthy cells was assessed using the CyQUANT Direct Cell Proliferation Assay (#C35011, Invitrogen, Carlsbad, CA, USA). Splenic T-cells from Pmel1-TCR transgenic mice (Jax stock #Stock No: 005023), which bear melanoma-specific gp100 epitope (KVPRNQDWL), were prelabeled with carboxylfluorescein succinimidyl ester (CFSE) dye, the dilution of which correlates tightly with an increase in cell proliferation, and activated for 3 days in the presence of gp100 peptide epitope (#AS-62589, AnaSpec, Fremont, CA, USA). T-cells kept in IL-2 for three days without TCR activation served as negative control. Next, TCR-activated T-cells were either placed in culture alone (positive control) or in the presence of tumor cells (RF420) at a 2:1 ratio for 3 days. In addition, some co-cultures were treated with a control IgG1 (10 µM) or with hSFRP2 mAb (10 µM). After 3 days, CD8+ T-cells from the co-cultures were used to measure CFSE intensity. Mean fluorescence intensity (MFI) was measured by FACS, and analysis was done using FlowJo software. Percent suppression was determined based on the division index, which is calculated by multiplying the proliferation index by the percentage of divided cells, and thus represents the division status of the entire population (34). The experiments were repeated three times.

### 2.16. Evaluation of Apoptotic Effects of hSFRP2 mAb

Splenocytes isolated from C57BL/6 mice were stimulated for 48 h in TCR (anti-CD3 (2 µg/mL), anti-CD28 (5 µg/mL) and 6000 U/mL IL-2 and then removed from the wells, washed twice with PBS, and CD4^+^/CD8^+^ T-cells were selected as described above. Selected T-cells were resuspended in the T-cell medium with 6000 U/mL IL-2 for 24 h and then treated with hSFRP2 mAb (10 µM), of IgG1 (10 μM) or left untreated for 24 h (*n* = 3). A positive control containing cells previously frozen and thawed in DMSO-containing media was run in parallel (*n* = 3). Cells were finally stained following the Promokine Apoptotic/Necrotic/Healthy Cells Detection Kit (#PK-CA707-33018) protocol using 5 μL of annexin-V, stained with Hoechst for 15 min, analyzed by flow cytometry on the LSRFortessa and analyzed with FlowJo v10 software (Tree Star, OR, USA) to identify double-positive cells.

### 2.17. Statistics

A priori power and sample size calculations were performed using PASS version 08.0.13. For continuous measures, 9 measures per condition yielded 82% power to detect a standardized effect size (mean difference divided by SD) of 1.45 based on a two-sample t-test with two-sided α = 0.05. This is a large effect size, but large differences are required in order to translate into clinically and biologically meaningful effects. In vitro experiments were performed in triplicate and repeated three times. Quantitative measures were collected with technician blinded to experimental conditions to mitigate potential bias. Group comparisons of continuous measures were performed using two-sample t-tests or ANOVA for two- or multi-group comparisons, respectively. Equivalent non-parametric tests (Wilcoxon rank-sum or Kruskal–Wallis tests) were used as appropriate. Comparisons of proportions were performed using Fisher’s exact test. Summary result values for continuous variables are presented as mean ± standard error of the mean. The statistical tests described above were performed using Stata v16.1, College Station, TX, USA). *p*-values of less than 0.05 were considered to be statistically significant.

## 3. Results

### 3.1. Humanized SFRP2 mAb Inhibits Tumor Metastases In Vivo

**Figure 1:**
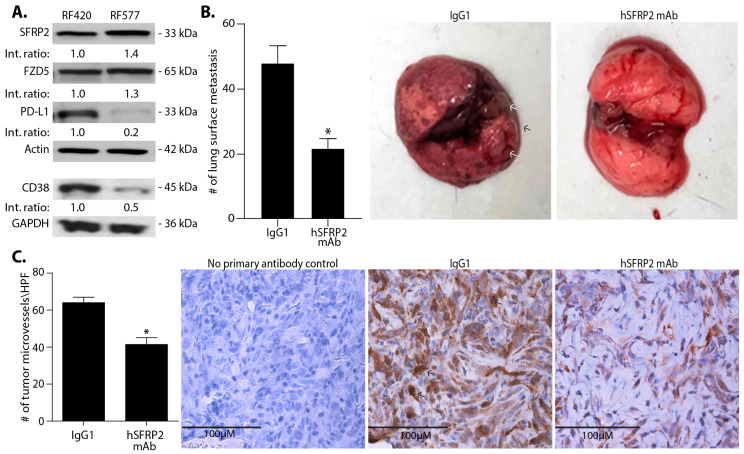
hSFRP2 mAb reduces metastatic osteosarcoma growth in vivo. (**A**) Characterization of two osteosarcoma cell lines: RF420 (left) and RF577 (right) by Western blot analysis probing for SFRP2, FZD5, CD38 and PD-L1 levels. Loading control: actin and GAPDH. Western blot shows SFRP2, FZD5, PD-L1 and CD38 proteins detected in both cell lines. PD-L1 and CD38 proteins were detected in RF420 with lower levels than in RF577. Int. ratio: intensity ratios listed below SFRP2, FZD5, PD-L1, and CD38 Western blots were calculated in RF577 cells after normalization to both the loading control (actin or GAPDH) and reference sample in RF420 cells (indicated by 1.00). (**B**) In vivo monotherapy with hSFRP2 mAb in RF420 OS lung metastasis model. Osteosarcoma RF420 cells were injected iv via the tail vein in C57BL/6 mice. Treatments with IgG1 control (4 mg/kg weekly) or hSFRP2 mAb (4 mg/kg every 3 days) started 8 days after tumor cell injection. After 21 days of treatment, animals were euthanized, lungs were resected and surface nodules were quantified. Left: quantification of lung metastases in IgG1-treated control mice (*n* = 19) versus hSFRP2 mAb treated mice (*n* = 22) (* *p* < 0.001). Middle: representative lungs from a mouse treated with IgG1 control with multiple OS tumor metastases (arrows). Right: representative lungs from a mouse treated with hSFRP2 mAb with minimal OS metastases (**C**) Angiogenesis of metastases analyzed on paraffin-embedded lungs that underwent immunohistochemistry with antibody to CD31. Left: quantification of microvessel density measured on microphotographs of metastases from CD31-stained tissues, showing a reduction in hSFRP2 mAb-treated tumors (*n* = 10, * *p* ≤ 0.001). Right: representative images of CD31-stained sections of lung tumors with negative control, IgG1 control-treated, and hSFRP2 mAb-treated. HPF: High Power Field. Scale bar: 100 µm.

To test the efficacy of SFRP2-targeting immunotherapy on metastatic osteosarcoma, we used two genetically engineered murine cell lines, RF420 and RF577 [17,18]. First, we measured SFRP2, FZD5, CD38 and PD-L1 protein levels by Western blot. Western blot detected SFRP2, FZD5, PD-L1 and CD38 protein in both cell lines. However, while the levels of PD-L1 and CD38 proteins were high in RF420 cells, they were lower in RF577 cells (Figure 1A).

To determine the time required for lung metastases to be established after tumor-cell inoculation in mice, RF420 tumor cells were injected via the tail vein of 3 C57BL/6 mice, the mice were sacrificed at different time points, and their lungs were evaluated by microscopy. On day 5 after the tail vein injection, lungs from one mouse were resected, and hematoxylin and eosin (H&E)-staining performed on paraffin-embedded lung sections revealed no metastases. On day 7 after injection, two mice were sacrificed, and both mice had visible lung metastases (Supplementary Materials Figure S1A). For RF577, four mice were injected via the tail vein, and two mice were sacrificed per time point on days 8 and 11. At day 8, there were no lung metastases, while, at day 11, both mice had lung metastases visualized by H&E (Supplementary Materials Figure S1B).

To test the effect of hSFRP2 mAb on metastases, RF420 cells were injected iv via the tail vein of male and female C57BL/6 mice in two independent experiments, and the results were combined. Mice were treated for 21 days, starting treatment on day 8, with either hSFRP2 mAb (4 mg/kg injected iv every three days; *n* = 22) or with IgG1 control (4 mg/kg injected iv every seven days; *n* = 19). The dose and frequency of the hSFRP2 mAb was chosen based on previously published MTD and PK studies [10]. During the course of these experiments, no mice in either treatment group exhibited weight loss, hair loss or lethargy. After euthanasia on day 21 of treatment, lungs were resected, and surface metastases were counted. The number of surface metastases was 47.8 ± 5.3 in the IgG1 (*n* = 19) control group, which was significantly reduced to 21.6 ± 3.0 in the hSFRP2 mAb treated group (*p* < 0.001, *n* = 22, Figure 1B). This demonstrates that hSFRP2 mAb is safe and effective as a monotherapy to inhibit metastatic osteosarcoma.

### 3.2. Tumor Angiogenesis Is Decreased in hSFRP2 mAb-Treated Lung Metastases

We previously showed that the hSFRP2 mAb reduces tumor angiogenesis in primary tumors in vivo [10]; however, this has not been evaluated in genetically engineered mouse models (GEMM). Paraffin-embedded RF420 lung metastases underwent IHC with a CD31 antibody, and microvascular density counts/HPF were 64.0 ± 5.04 in the control group and reduced to 41.4 ± 6.29 in hSFRP2 mAb-treated group (*n* = 10, *p* < 0.001, Figure 1C), demonstrating that hSFRP2 mAb inhibits tumor angiogenesis in a GEMM model.

### 3.3. The Combination of hSFRP2 mAb and PD-1 mAb Is Additive in Inhibiting Metastatic Osteosarcoma Growth In Vivo

**Figure 2:**
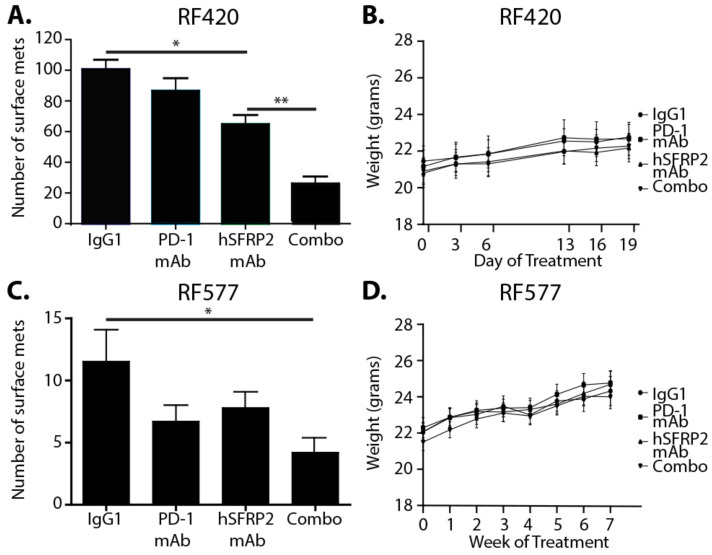
hSFRP2 mAb is additive to mPD-1 mAb in reducing metastatic osteosarcoma growth in vivo. Two models of OS lung metastases using RF420 or RF577 cell lines were generated by tail vein injection in C57BL/6 mice. (**A**,**B**) For the metastatic RF420 osteosarcoma model, treatments started eight days after tumor cell injection with either IgG1 control (4 mg/kg, iv, weekly; *n* = 12), mPD-1 mAb (200 ug/100 µL ip, q 3 days, *n* = 10), hSFRP2 mAb (4 mg/kg iv, q 3 days, *n* = 12) or a combination of both treatments (*n* = 11). (**A**) After three weeks of treatment, mice were euthanized and lungs removed. High-resolution photographs were taken and utilized to quantitate metastatic surface lung nodules for each treatment group. Compared to the IgG1 control treatment, there was no significant reduction in surface metastases with PD-1 mAb alone (*p* = 0.577). hSFRP2 mAb reduced surface metastases significantly compared to the control (* *p* = 0.001). The combination therapy resulted in a significant reduction of surface nodules compared to hSFRP2 mAb treatment (** *p* < 0.0001). (**B**) Weights of all mice were measured and recorded weekly. There was neither a significant reduction in weight during treatment nor significant differences in weight between the groups during treatment. (**C**,**D**) Metastatic RF577 osteosarcoma in vivo experiment using the same four treatment regimens as described in the RF420 model. Treatments began at day 12 and continued for 49 days. (**C**) High resolution images of the extracted lungs were taken for each treatment group: IgG1 (*n* = 13), mPD-1 mAb (*n* = 14), hSFRP2 mAb (*n* = 15) and a combination (*n* = 14), and utilized to quantify lung surface nodules. Combination therapy significantly decreased lung surface metastasis compared to the IgG1 control (* *p* = 0.018). (**D**) Animal weights were measured starting on the first day of treatment and then weekly until the final week of treatment. No significant reduction in weight was seen in any treatment group.

A phase II clinical trial showed no effect of nivolumab on metastatic osteosarcoma [12]. To evaluate whether the hSFRP2 mAb could overcome PD-1 mAb primary resistance, RF420 cells were injected via the tail vein of male and female C57BL/6 mice (22 males and 23 females). On day 8 after RF420 cell injection, mice were treated with either 4 mg/kg iv IgG1 control weekly (*n* = 12), 8 mg/kg ip murine anti-PD-1 mAb (mPD-1 mAb) every 3 days (*n* = 10), 4 mg/kg iv hSFRP2 mAb every 3 days (*n* = 12) or the combination of both 4 mg/kg iv hSFRP2 mAb every 3 days and 8 mg/kg ip mPD-1 mAb given on the same treatment day (*n* = 11). After 21 days of treatment, mice were euthanized, lungs were harvested, and the number of surface metastases was counted in each group. The number of surface metastases was: 100.6 ± 6.3 for the IgG1 control, 64.8 ± 4.6 for hSFRP2 mAb, 86.6 ± 8.2 for mPD-1 mAb and 24.2 ± 4.6 for hSFRP2 mAb + mPD-1 mAb (Figure 2A). For monotherapy, the number of surface metastases was significantly reduced in the hSFRP2 mAb-treated group compared to the IgG1 control group (*p* = 0.001), whereas mPD-1 mAb did not show any significant antimetastatic activity compared to the control. The therapeutic combination of mPD-1 mAb and hSFRP2 mAb significantly reduced the number of lung surface metastases compared to hSFRP2 mAb monotherapy (*p* < 0.0001; Figure 2A). These results were confirmed by counting metastases on H&E stained lung sections. Compared to IgG1 control treatment, there was no significant reduction in surface metastases with PD-1 mAb alone (*p* = 0.69). hSFRP2 mAb reduced surface metastases significantly compared to the control (*p* = 0.0031). The combination therapy resulted in a significant reduction of surface nodules compared to hSFRP2 mAb treatment (** *p* < 0.0001) (Supplementary Materials Figure S2). There was no weight loss, hair loss or lethargy observed in any of the treatment groups during the course of the experiment (Figure 2B).

To investigate whether hSFRP2 mAb monotherapy and a combined therapy with mPD-1 mAb would be effective in a second osteosarcoma GEMM model, RF577 OS cells were injected via the tail vein into C57BL/6 male and female mice. Mice were treated either with IgG1 control (*n* = 14), mPD-1 mAb (*n* = 15), hSFRP2 mAb (*n* = 16) or a combination of both immunotherapies (*n* = 14) for 49 days. Three mice were excluded from analyses (1 IgG1 control mouse was found dead on day 43 of treatment and on autopsy had numerous surface lung metastases; 1 mPD-1 mAb-treated mouse was euthanized on day 32 for labored breathing and had 2 surface metastases, and 1 hSFRP2 mAb-treated mouse was euthanized on day 39 and had 2 surface metastases. These latter two mice had been involved in fights). Of the 56 remaining mice analyzed (13 animals treated with IgG1 control, 14 animals treated with mPD-1 mAb, 15 animals treated with hSFRP2 mAb and 14 treated with combination immunotherapy) on day 49 of treatment, the number of surface metastases was 11.5 ± 2.5 in the IgG1-treated group, 6.7 ± 3 in the mPD-1 mAb-treated group, 7.8 ± 1.3 in the hSFRP2 mAb-treated group and, in the combination therapy group, was 4.2 ± 1.1 (*p* = 0.018, comparing IgG1 versus combination; Figure 2C). There was no weight loss (Figure 2D), hair loss or lethargy in any of the remaining mice.

### 3.4. Serum SFRP2 Is Elevated in Tumor-Bearing Mice and Responds to Therapy

**Figure 3:**
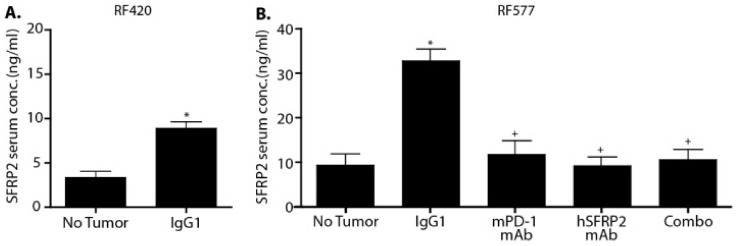
Serum SFRP2 levels are elevated in mice with metastatic osteosarcoma compared to non-tumor-bearing mice and responds to therapy. (**A**) Serum levels of SFRP2 were compared between C57BL/6 mice with metastatic RF420 OS treated with IgG1 control and non-tumor-bearing C57BL/6 mice with ELISA. There were significantly higher levels of SFRP2 in the serum of mice with metastatic RF420 OS versus mice without tumors (*n* = 3, * *p* < 0.01). (**B**) ELISA was used to compare the serum levels of SFRP2 in all treatment groups of the C57BL/6 mice with metastatic RF577 OS and C57BL/6 mice without tumors. The serum level of SFRP2 was significantly higher in the group of tumor-bearing mice treated with IgG1 compared to non-tumor-bearing mice (* *p* < 0.01) (*n* = 9 and *n* = 8, respectively). The serum level of SFRP2 was significantly decreased in the mPD-1 mAb (*n* = 8), hSFRP2 mAb (*n* = 12) and Combo (*n* = 12) treatment groups compared to IgG1-treated mice (^+^ *p* < 0.01).

Serum SFRP2 has been shown to be elevated in patients with breast cancer compared to patients without cancer [19]. To evaluate whether SFRP2 is elevated in blood from mice with metastatic osteosarcoma compared to mice without tumors, mouse serum from RF420 tumor-bearing mice was drawn at the end point (day 27), and SFRP2 protein levels were compared to the levels in non-tumor-bearing mice. The serum concentration of SFRP2 was increased in control tumor-bearing mice (8.91 ± 0.67 ng/mL), compared to non-tumor-bearing mice (3.36 ± 0.74 ng/mL, *n* = 3; *p* < 0.01; Figure 3A). Similarly, in the RF577 model, the levels of the SFRP2 protein were increased in the serum of control tumor-bearing mice (*n* = 9) compared to non-tumor-bearing mice (32.6 ± 2.64 ng/mL versus 9.30 ± 2.52 ng/mL, respectively; *p* < 0.01; *n* = 8, Figure 3B). In addition, we compared the levels of SFRP2 between the treatment groups: control (*n* = 9), mPD-1 mAb (*n* = 8), hSFRP2 mAb (*n* = 12) and combination therapy (*n* = 12). All treatment groups had significantly lower SFRP2 levels compared to IgG control-treated mice (32.6 ± 2.64 ng/mL for IgG control, 11.7 ± 3.12 ng/mL for mPD-1 mAb, 9.14 ± 2.02 ng/mL for hSFRP2 mAb and 10.5 ± 2.30 ng/mL for combination; *p* < 0.01; Figure 3B).

### 3.5. Immune Activation and Exhaustion Marker Levels Are Altered in T-Cells from Mice Treated with hSFRP2 mAb

**Figure 4:**
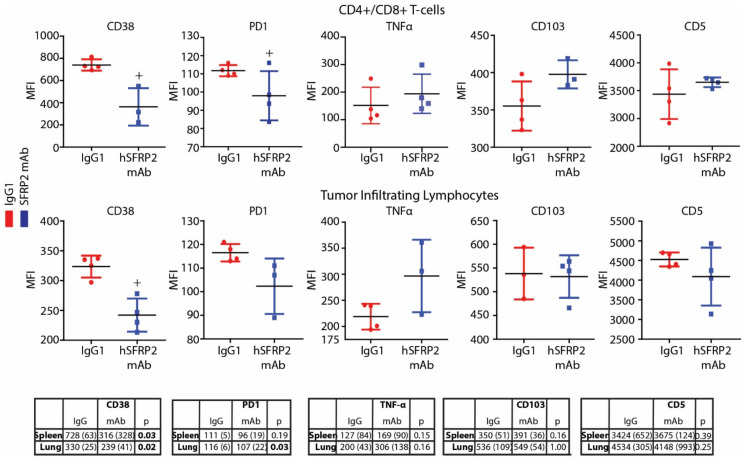
hSFRP2 mAb reduces T-cell CD38 and PD-1 protein in vivo. Splenic CD4^+^ and CD8^+^ T-cells (**top**) and tumor infiltrating lymphocytes (TILs; **bottom**) isolated from RF420 tumor-bearing mice treated with IgG1 control (*red*) or hSFRP2 mAb (*blue*) for 21 days were stained for the following immune markers labeled with fluorochromes: CD38, PD-1, TNF-α, CD103 and CD5. Dot plot graphs show mean fluorescent intensity (MFI) analyzed by FACS for each of the immune markers of interest within the CD4^+^/CD8^+^ and TIL populations. For IgG1 and hSFRP2 mAb treatments, the number of independent samples (n) was 4. CD38 was significantly decreased with hSFRP2 mAb in both splenocytes (+ *p* < 0.05) and TILs (+ *p* < 0.05). PD-1 was significantly decreased in CD8^+^ TILs treated with hSFRP2 mAb (+ *p* < 0.05). Bottom tables: median MFIs (interquartile range) for each immune marker.

To determine whether hSFRP2 mAb therapy alters the immune response, tumor-infiltrating lymphocytes (TILs) and splenic T-cells from RF420 tumors from mice treated with IgG1 control or hSFRP2 mAb for 21 days were analyzed by flow cytometry for cell surface markers. A FACs analysis demonstrated that compared to the IgG1 control group, CD38 was significantly decreased in mice treated with hSFRP2 mAb in both splenic T-cells (median (interquartile range): 316 (328) for hSFRP2 mAb versus 728 (63) for IgG1; *n* = 4; *p* = 0.03) and TILs (median (interquartile range): 239 (41) for hSFRP2 mAb versus 330 (25) for IgG1; *n* = 4; *p* = 0.02) (Figure 4). PD-1 was significantly reduced in TILs from mice treated with hSFRP2 mAb, compared to the IgG1 control group (median (interquartile range): 107 (22) for hSFRP2 mAb versus 116 (6); *n* = 4; *p* = 0.03). Other representative T-cell exhaustion markers, such as CD103, TNFα or CD5, were not significantly affected by hSFRP2 mAb treatment in splenocytes or TILs (*n* = 4, *p* = NS).

**Figure 5:**
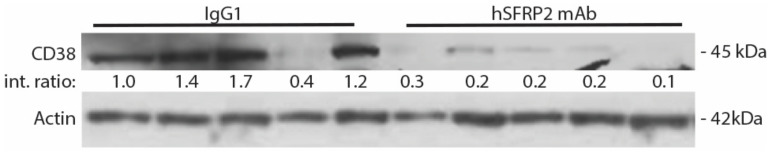
Treatment with hSFRP2 mAb in mice with metastatic osteosarcoma decreases CD38 protein levels in splenocytes. Splenocytes isolated from mice with RF577 OS lung metastases treated with IgG1 control (*n* = 5) versus hSFRP2 mAb (*n* = 5) were lysed and prepared for Western blot analysis probing for CD38. Int. ratio: intensity ratios listed below CD38 Western blot were calculated after normalization to both actin and the reference sample (indicated by 1.00). Overall, CD38 protein levels were reduced in splenocytes from mice treated with hSFRP2 mAb by 82%, compared to the control group (*p* = 0.004).

To test if the reduction in CD38 protein levels induced by hSFRP2 mAb treatment occurs in a second cell line in vivo, splenocytes isolated from mice with RF577 OS tumors were subjected to Western blot, probing for CD38. The mean relative CD38 protein levels normalized to actin were reduced in splenocytes from mice treated with hSFRP2 mAb by 82% compared to the control group (*p* = 0.004, *n* = 5 per group; Figure 5).

### 3.6. SFRP2 Increases CD38 and PD-1 in T-Cells via NFATc3

**Figure 6:**
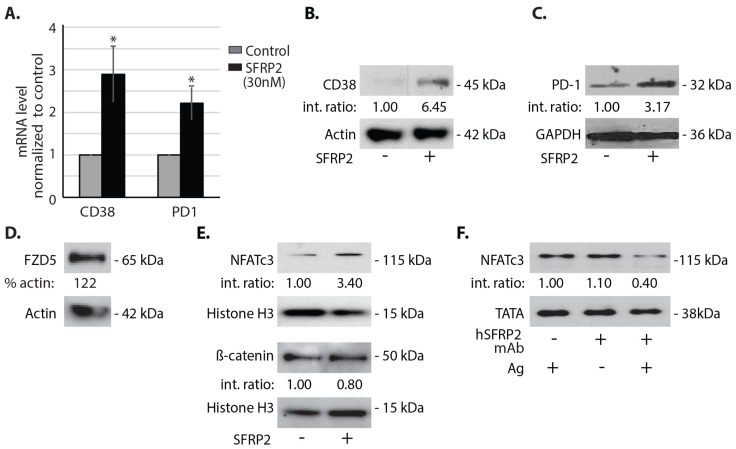
SFRP2 increases CD38 and PD-1 mRNA and protein levels in T-cells. (**A**) Splenic T-cells cultured in IL2-containing medium were treated with or without SFRP2 (30 nM) for 1 h, and the mRNA levels for CD38 and PD-1 were measured by qRT-PCR (*n* = 8). GAPDH was used as an internal control. (**B**–**F**) All T-cells were treated with IL-2. Samples were probed using Western blot with antibodies to the indicated protein markers. Actin was the loading control for cytoplasmic fraction; histone H3 and TATA were loading controls for nuclear fractions. (**B**,**C**,**E**,**F**) Int. ratio: intensity ratios listed below Western blots for (**B**–**F**) were calculated after normalization to both loading control and reference samples (indicated by 1.00). (**B**,**C**) Cytoplasmic fractions of wild-type splenic T-cells were isolated and protein levels of CD38 (**B**) and PD-1 (**C**) were increased in SFRP2-treated T-cells, compared to untreated cells. (**D**) Western blot probing for FZD5 protein in mouse splenic T-cells. % actin: total band intensity was normalized to actin. (**E**) Splenic T-cells were untreated or treated with recombinant SFRP2 protein for 1 h. The nuclear fraction was isolated from the T-cells, and nuclear NFATc3 and β-catenin protein levels were measured. Nuclear fractions demonstrated increased NFATc3 protein levels but no change in β-catenin levels with SFRP2 treatment. (**F**) Splenic T-cells were activated with antigen gp100 (0.87 μM) with or without 10 µM of hSFRP2 mAb treatment. Nuclear fractions demonstrated decreased NFATc3 protein levels in hSFRP2 mAb-treated cells activated by antigen.

Since T-cells isolated from mice treated with hSFRP2 mAbexpress lower levels of CD38 and PD-1 proteins than T-cells isolated from control mice, we asked whether SFRP2 regulates CD38 and PD-1 expression in T-cells. First, we stimulated splenic T-cells with SFRP2 (30 nM) for 1 h and measured the levels of CD38 and PD-1 mRNA in SFRP2-treated T-cells, compared to control T-cells (Figure 6A). SFRP2 stimulation increased CD38 mRNA levels by 2.9 ± 0.64 times (*n* = 8, *p* = 0.015) and PD-1 mRNA levels by 2.2 ± 0.37 times (*n* = 8, *p* = 0.008). Next, we asked whether the increase in CD38 and PD-1 mRNA levels was accompanied by an increase of CD38 and PD-1 protein levels (Figure 6B,C). Upon SFRP2 stimulation (30 nM), CD38 protein levels increased 6.45-fold (Figure 6B), and PD-1 protein levels increased 3.17-fold (Figure 6C).

In endothelial and tumor cells, SFRP2 binds to the FZD5 receptor and stimulates the calcineurin/NFATc3 pathway. Activated NFATc3 then translocates from the cytoplasm into the nucleus and stimulates angiogenesis. SFRP2-induced angiogenesis and tumor growth is not mediated through β-catenin [15]. Whether SFRP2 utilizes a similar signaling pathway in T-cells has not previously been evaluated. First, Western blot on lysates from splenic T-cells showed FZD5 protein to be present (Figure 6D). To evaluate whether SFRP2 activates NFATc3 or β-catenin signaling in T-cells, splenic T-cells were treated with recombinant SFRP2 protein (30 nM) for 1 h, then nuclear and cytoplasmic fractions were isolated, and proteins were extracted and analyzed by Western blot probing for NFATc3 and β-catenin. Nuclear NFATc3 increased 3.4-fold after stimulation with SFRP2 recombinant protein compared to control (Figure 6E). Comparatively, the levels of nuclear β-catenin remained unchanged under the same conditions (Figure 6E). This demonstrates that SFRP2 activates NFATc3 in T-cells with no effect on β-catenin.

Next, splenic T-cells were stimulated with or without antigen gp100 to activate NFATc3 and treated with or without hSFRP2 mAb (10 µM). Lysates from nuclear fractions were then analyzed by Western blot. NFATc3 protein was present in the nuclear fraction of antigen-stimulated T-cells (Figure 6F). hSFRP2 mAb had no effect on NFATc3 in the absence of antigen, however, NFATc3 levels decreased by 50% in antigen-activated T-cells treated with hSFRP2 mAb (Figure 6F). This suggests that hSFRP2 mAb inhibits NFATc3 only when it is induced by antigen but has no effect on baseline levels of NFATc3 in quiescent T-cells.

### 3.7. Humanized SFRP2 mAb Inhibits TGFβ-Induced Elevation of CD38 and PD-1 in T-Cells

**Figure 7:**
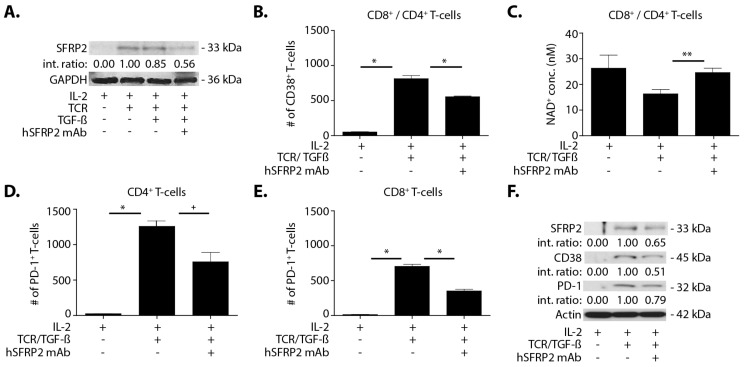
Humanized SFRP2 mAb inhibits TCR/TGFβ-induced elevation of CD38 and PD-1 in T-cells. (**A**) Western blot showing the levels of SFRP2 protein in T-cells treated either with IL-2 (6000 U/mL) alone, IL-2 + TCR, IL-2 + TCR + TGFβ (5 ng/mL) or IL-2 + TCR + TGFβ + hSFRP2 mAb (30 nM). Loading control: GAPDH. Int. ratio: intensity ratios listed below SFRP2 Western blot were calculated after normalization to both GAPDH and the reference sample (indicated by 1.00). T-cell activation by TCR/TGFβ increases the levels of SFRP2 protein, which are decreased by hSFRP2 mAb treatment. (**B**) FACS analysis showing the levels of CD38 in CD8^+^/CD4^+^ T-cells after incubation with IL-2 alone, IL-2 + TCR/TGFβ or IL-2 + TCR/TGFβ + hSFRP2 mAb. CD38 protein levels increase upon activation by TCR/TGβ, compared to control (*n* = 3, * *p* < 0.001), and this effect is abrogated by treatment with hSFRP2 mAb (*n* = 3, * *p* < 0.001). (**C**) NAD^+^ concentration was measured using an NAD^+^/NADH cell-based assay kit on T-cells exposed to IL-2 alone, IL-2 + TCR + TGFβ or IL-2 + TCR + TGFβ + hSFRP2 mAb. T-cell activation by TCR/TGβ led to a decrease in NAD^+^ concentration compared to IL-2, which was restored by hSFRP2 mAb treatment (*n* = 3, ** *p* = 0.02). PD-1 levels were measured by FACS in (**D**) CD4^+^ and (**E**) CD8^+^ T-cells exposed to IL-2 alone, IL-2 + TCR + TGFβ or IL-2 + TCR + TGFβ + hSFRP2 mAb. In both cell types, T-cell activation by TCR/TGFβ induced an increase in PD-1 protein levels (*n* = 3, * *p* < 0.001), which was reduced by hSFRP2 mAb treatment (*n* = 3, ^+^ *p* <0.05) in CD4^+^ cells (**D**) and *n* = 3, * *p* < 0.001 in CD8^+^ cells (**E**). (**F**) Results obtained by FACS analysis on T-cells exposed to IL-2 alone, IL-2 + TCR + TGFβ or IL-2 + TCR + TGFβ + hSFRP2 mAb were confirmed by Western blot for the indicated markers. TCR/TGFβ activation led to an increase in SFRP2, CD38 and PD-1 protein levels, which were reduced by hSFRP2 mAb treatment. Loading control: actin. Int. ratio: intensity ratios listed below SFRP2, CD38 and PD-1 Western blots were calculated after normalization to both actin and the reference sample (indicated by 1.00).

In the tumor microenvironment, CD38 and PD-1 in T-cells are upregulated by TGFβ [20]. Given the importance of TGFβ in the microenvironment, whether T-cell activation by TCR and TGFβ affects the SFRP2 protein level was investigated. The activation of T-cells by TCR increased SFRP2 protein levels by Western blot, but these levels were not further increased by the addition of TGFβ. The TCR/TGFβ-stimulated SFRP2 increase was prevented by hSFRP2 mAb (Figure 7A). A FACS analysis from this same experiment further revealed a statistically significant elevation in CD38 positive T-cells with the addition of TCR/TGFβ (from 53.3 ± 1.8 cells with IL-2 to 813 ± 44.1 cells with IL-2 + TCR/TGFβ; *n* = 3; *p* < 0.001), and this was reduced by the hSFRP2 mAb (from 813 ± 44.1–555.3 ± 8.6 cells; *n* = 3; *p* < 0.001, Figure 7B), highlighting the contribution of SFRP2 in CD38 upregulation by TGFβ.

The upregulation of CD38 decreases NAD+, resulting in conditions that promote cancer growth [20]; therefore, we evaluated the effect of hSFRP2 mAb on NAD+. T-cells treated with TCR/TGFβ led to a decrease in NAD+ levels compared to control (control 26.3 ± 5.1 nM, TCR/TGFβ 16.3 ± 1.7 nM (*n* = 3, *p* = 0.13)). NAD+ levels were statistically significantly increased by treating T-cells with hSFRP2 mAb (24.7 ± 1.7 nM, *n* = 3; *p* = 0.02, Figure 7C). This demonstrates that SFRP2 plays a role in CD38 and NAD+/NADH regulation by TGFβ.

To further demonstrate that PD-1 is affected by SFRP2 inhibition, PD-1 levels were analyzed by flow cytometry in CD4+ and CD8+ T-cells. TCR/TGFβ stimulation increased the number of PD-1 positive T-cells from 25.0 ± 0.6–1259.7 ± 74.3 cells in the CD4+ population (Figure 7D; *n* = 3; *p* < 0.001) and from 14 ± 2.1–705 ± 30.4 cells in the CD8+ (Figure 6E; *n* = 3; *p* < 0.001). The treatment of TCR/TGFβ-stimulated T-cells with hSFRP2 mAb significantly reduced PD-1 protein levels in both CD4+ cells (from 1259.7 ± 74.3 cells with TCR/TGFβ to 758.7 ± 130.7 with TCR/TGFβ + hSFRP2 mAb; *n* = 3; *p* < 0.05, Figure 7D) and CD8+ cells (from 705 ± 30.4 cells with TCR/TGFβ to 353 ± 23.3 with TCR/TGFβ + hSFRP2 mAb; *n* = 3; *p* < 0.001, Figure 7E).

To confirm the results obtained through the FACS analysis, the effects of TCR/TGFβ and hSFRP2 mAb treatment on T-cells were analyzed for changes in SFRP2, CD38 and PD-1 protein levels by Western blot. All three proteins were undetectable in naïve T-cells (Figure 7F). However, the activation of T-cells by TCR/TGFβ led to an increase in SFRP2, CD38 and PD-1 protein levels, which was inhibited by hSFRP2 mAb (SFRP2 protein reduced by 45%, CD38 protein by 49% and PD-1 by 21% (Figure 7F). In conclusion, quiescent T-cells do not synthesize SFRP2 protein, while activated T-cells produce SFRP2 protein. The regulation of CD38 and PD-1 production by TGFβ in T-cells can be antagonized by hSFRP2 mAb.

### 3.8. Humanized SFRP2 mAb Rescues T-Cell Proliferation Inhibition Tumor Cells In Vitro

**Figure 8:**
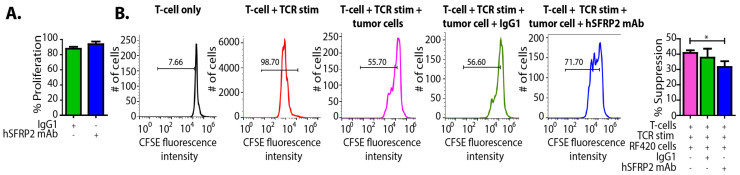
Tumor-induced suppression of T-cells is reversed with hSFRP2 mAb treatment. (**A**) RF420 cells were plated at 3000 cells/well and incubated with either IgG1 control (10 µM) or hSFRP2 mAb (10 µM) for 72 h (*n* = 6). The number of healthy cells was assessed using Cyquant Direct Cell Proliferation Assay. This shows that hSFRP2 mAb does not directly affect RF420 cell viability. (**B**) T-cells were prelabeled with CFSE dye and then cultured for three days alone, with TCR-stimulation or in the presence of RF420 cells, under the following conditions: T-cells alone (far left, *black*); anti-CD3 and anti-CD28 antibodies (TCR) (*red*); TCR and co-culture with RF420 cells (*pink*); TCR stimulated T-cells co-cultured with RF420 cells that were treated with either 10 µM IgG1 control (*green*) or 10 µM of hSFRP2 mAb (*blue*). Intensity was measured for each condition using a FACS analysis. Stimulation with TCR alone was used as a positive control for this experiment. *Bar graph*: percent suppression of CD8^+^ T-cells was calculated based on the division index method. The division index is calculated by multiplying the proliferation index by the percentage of divided cells and thus represents the division status of the entire population. The experiments were repeated thrice. The histograms display representative analyses of CD8^+^ T-cells, while the bar graph represents cumulative data from all repeats (*n* = 3, * *p* < 0.05).

In cancer patients, altered T-cell immune function is blunted by factors tumors secrete in the environment. Several tumor cell lines produce soluble factors that inhibit T-cell proliferation [21]. Thus, whether antagonizing SFRP2 could overcome the tumor-induced inhibition of T-cell proliferation was investigated. First, a direct effect of hSFRP2 mAb on reducing tumor proliferation was evaluated by incubating RF420 osteosarcoma cells with either IgG1 control (10 µM) or hSFRP2 mAb (10 µM) for 72 h (*n* = 6). There was no difference in tumor cell proliferation between groups (Figure 8A). Next, T-cells were incubated for 72 h either alone, with TCR stimulation, with TCR stimulation + RF420 cells, with TCR stimulation + RF420 cells + IgG1 control (10 µM) or with TCR stimulation + R420 cells + hSFRP2 mAb (10 µM) (*n* = 3 per group). Compared to the proliferation of quiescent T-cells alone (7.66%), the TCR antigen (positive control) increased the proliferation index to 98.7%. This percentage decreased to 55.7% when TCR-stimulated T-cells were cultured with RF420 cells, confirming the inhibitory effect of tumor cells on T-cell proliferation. The addition of the IgG1 control to the co-culture had no effect (55.6% with IgG1 versus 55.70 without). However, the addition of hSFRP2 mAb in the co-cultures partially rescued T-cell proliferation with an increase to 71.7%. Overall, the percentage of suppression of proliferation decreased significantly from 40.8 ± 1.0% when TCR-stimulated T-cells were incubated with tumor cells, to 31.6 ± 2.16% when hSFRP2 mAb was added to the co-cultures (*n* = 3; *p* < 0.05).

### 3.9. Treatment with hSFRP2 mAb Increases the Apoptosis of Tumor Cells, but Has No Effect on T-Cell Apoptosis

**Figure 9:**
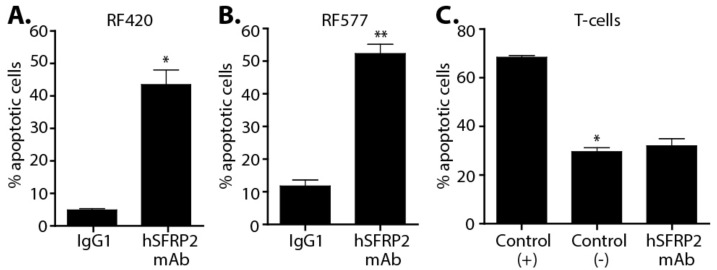
Impact of hSFPR2 mAb treatment on osteosarcoma and T-cell apoptosis. (**A**,**B**) Apoptosis of RF420 (**A**) and RF577 (**B**) OS tumor cells was measured after treatment for 1 h with 10 µM IgG1 control or hSFPR2 mAb using an apoptosis detection kit allowing the identification of FITC^+^ apoptotic cells. The percentage of apoptotic cells after treatment with hSFRP2 mAb increased significantly. (**A)** In RF420 cells, apoptosis increased from 4.9 0.19% in IgG1 treated cells to 43.54 0.16% in cells treated with hSFRP2 mAb (*n* = 12, * *p* < 0.001) and, (**B)** in RF577 cells, hSFRP2 mAb increased apoptosis to 52.4 0.08% from 11.8 0.3% in IgG-treated cells (*n* = 12, ** *p* < 0.0001). (**C**) Apoptosis in CD4^+^ and CD8^+^ T-cells. T-cells were isolated from C57BL/6 mouse spleens, treated with IgG1 (10 µM) or hSFRP2 mAb (10 µM) for 24 h, stained with Hoechst and Annexin V and then analyzed by flow cytometry. A positive control for apoptosis was obtained from T-cells passed through various freeze/thaw cycles in DMSO-containing medium was used for the experiment. The percentage of apoptotic cells in the IgG1-treated control samples was significantly lower than in the positive control group (*n* = 3, * *p* < 0.001). Compared to the IgG1-treated samples, the percentage of apoptotic cells remained unchanged in the hSFRP2 mAb-treated samples (*n* = 3, *p* = NS).

Antagonizing SFRP2 has previously been shown to induce apoptosis in endothelial cells and breast tumor cells [16], but the effects on T-cells have not been evaluated. RF420 and RF577 OS cells were cultured in vitro with hSFRP2 mAb (10 µM) or with IgG1 control (10 μM) for 1 h, and the percentage of apoptotic cells was quantified. hSFRP2 mAb significantly increased the apoptosis of RF420 OS cells from 4.9 ± 0.19% with IgG1 treatment to 43.54 ± 0.16% with hSFRP2 mAb treatment (*n* = 12, *p* < 0.001, Figure 9A). A similar response was observed in RF577 OS cells with apoptosis increasing from 11.8 ± 0.3% after treatment with IgG1 to 52.4 ± 0.08% for RF577 cells treated with hSFRP2 mAb (*n* = 12, *p* < 0.0001, Figure 9B). The effect of hSFRP2 mAb on T-cells was then examined. CD4+ and CD8+ T-cells were isolated from mouse spleens and then incubated with hSFRP2 mAb (10 μM) or IgG1 (10 μM) for 24 h. A positive control was added by freezing and thawing T-cells in DMSO. The percentage of apoptotic cells in the positive control was significantly higher compared to IgG1 control-treated cells (*n* = 3, *p* < 0.001; Figure 9C). However, there was no difference between IgG1-control and hSFRP2 mAb-treated T-cells in term of apoptosis (*n* = 3, *p* = NS, Figure 9C). Thus, the apoptotic effect mediated by hSFRP2 mAb selectively affects tumor cells but not T-cells.

## 4. Discussion

Secreted frizzled-related protein 2, a secreted protein involved in the non-canonical WNT calcineurin/ NFAT pathway in endothelial cells [6], is a validated therapeutic target for cancer. In the present study, we found that SFRP2 also activates the NFATc3 pathway in T-cells, with no effect on β-catenin. SFRP2 stimulates angiogenesis, is antiapoptotic and has been shown to contribute to tumor growth in breast cancer [16,22,23,24], breast cancer lung metastases [25], angiosarcoma [16], rhabdomyosarcoma [26], alveolar soft part sarcoma [27], malignant glioma [28], multiple myeloma [29], renal cell carcinoma [30], prostate cancer [31], lung cancer [32] and melanoma [33,34]. Additionally, growing evidence strongly supports the contribution of SFRP2 to osteosarcoma metastases [18]. The high expression of SFRP2 in OS patient samples correlates with poor survival and SFRP2 overexpression suppresses normal osteoblast differentiation, promotes OS features and facilitates angiogenesis. Functional studies revealed that the stable overexpression of SFRP2 within localized human and mouse OS cells significantly promoted cell migration and invasion in vitro and enhanced metastatic potential in vivo [5]. Additional studies knocking down SFRP2 within metastatic OS cells showed decreased cell migration and invasion abilities in vitro, thus validating a critical biological function carried out by SFRP2 [1]. In p53 mutation-associated OS models, SFRP2 overexpression was associated with an induction of *FOXM1* and *CYR61* oncogenes in a β-catenin-independent manner [5]. This compelling evidence supports the emergence of SFRP2 as a potential therapeutic target for metastatic osteosarcoma.

Our laboratory previously reported the development of a hSFRP2 mAb that binds to SFRP2 with high affinity, is well tolerated and is efficacious at inhibiting human triple-negative breast cancer and murine angiosarcoma growth in vivo [10]. Murine and human SFRP2 proteins are 98% homologous, and the peptide sequence targeted by the humanized antibody is 100% homologous between mouse and human SFRP2 [16]. Therefore, the antibody can be used in both human and mouse tumor models. The present study establishes that hSFRP2 mAb is an effective treatment for murine metastatic OS as a monotherapy using two OS metastatic models. Based on this data, the FDA granted rare pediatric disease designation for the hSFRP2 mAb for osteosarcoma [35]. The observed reduction in the number of lung surface nodules is accompanied by a decrease in microvessel density and an increase in tumor cell apoptosis, which is consistent with other tumor types that we have previously tested [10].

A noteworthy finding in this study is that hSFRP2 mAb treatment leads to a reduction in CD38 levels in TILs and CD4+ and CD8+ T-cells, and a reduction of PD-1 in TILS. This was validated in cultured T-cells in vitro in which SFRP2 directly upregulated both CD38 and PD-1 mRNA and protein levels. CD38 regulates T-cell metabolism through NAD+, and increasing CD38 levels results in a decrease in NAD+ concentration. Intracellular NAD+ levels have a profound influence on diverse signaling and metabolic pathways in T-cells, and hence dictate their functional fate. The CD38-NAD+ axis also plays a crucial role in altering T-cell response in various pathophysiological conditions [36]. We found that the hSFRP2 mAb not only reduced CD38 protein levels but also increased NAD+ production in T-cells. In the tumor microenvironment, CD38 and PD-1 in T-cells are upregulated by TGF-β [20]. We observed that SFRP2 protein levels increased in T-cells treated with TGF-β and that TGF-β-stimulated CD38 and PD-1 protein levels were reduced in hSFRP2 mAb-treated T-cells. This suggests that SFRP2 modulates the induction of CD38 and PD-1 by TGF-β in T-cells. Additionally, the tumor-induced suppression of T-cells was reversed by hSFRP2 mAb treatment in a co-culture model. This provides evidence that SFRP2 antagonism not only results in the inhibition of angiogenesis and the induction of tumor apoptosis, as previously reported [6,7], but also demonstrates immunotherapeutic activity.

One recently reported mechanism of resistance to checkpoint inhibitors is through the upregulation of CD38 [13]. Our finding that hSFRP2 mAb reduces CD38 in TILs provides the mechanistic rationale for combination therapy using hSFRP2 mAb with a checkpoint inhibitor. In the present study, we found that the hSFRP2 mAb had an additive effect, inhibiting metastatic osteosarcoma when combined with a PD-1 mAb, with no signs of toxicity. CD38 is widely expressed in humans and is found in leukocytes, platelets, erythrocytes, immature cells of the bone marrow, neuronal cells and glial cells of the central nervous system, peripheral nerves, skeletal muscle cells, cardiac muscle cells, pancreas islet cells, osteoclasts and bronchial epithelium [37]. Thus, CD38 presents both numerous drug design challenges and opportunities. Several combinations of checkpoint inhibitors with CD38 monoclonal antibodies have started in recent years and were temporarily placed on partial clinical hold by the FDA in October 2017 for evaluation of toxicity with final results not yet published [38]. In contrast to CD38, which is highly expressed in normal tissue, SFRP2 protein levels are low in normal tissue and higher in tumors [22,39]. As an example in vivo, SFRP2-targeted molecular imaging shows that SFRP2 is highly expressed in tumor vessels with low uptake in adjacent normal vessels or renal vessels [39]. According to data in the Human Genome Database, the protein expression of SFRP2 is low in normal tissue of the body, compared to CD38 [40,41]. Additionally, in this paper we found that only activated T-cells produce SFRP2 protein, and the hSFRP2 mAb only inhibits NFATc3 in activated T-cells. Therefore, targeting SFRP2 to lower CD38 levels in TILs and in T-cells could potentially lower the toxicity of combination therapies involving a PD-1 inhibitor. This concept is emphasized by our preclinical studies in immunocompetent animal models showing that treatment with hSFRP2 mAb as monotherapy or in combination with PD-1 inhibitors had no clinical toxicity.

The combination of hSFRP2 mAb and PD-1 mAb was additive in the RF20 OS cell line that had higher tumor PD-L1 protein as well as the RF577 OS tumor that had lower PD-1 protein. There are previous reports in other tumor types of PD-l1 negative patients that still respond to PD-1 inhibitors. One hypothesis for this is that the T-cells in the microenvironment may be PD-L1 positive. This requires further investigation in the future to determine if PD-L1 negative OS patients would benefit from combination therapy with hSFRP2 mAb [42].

hSFRP2 mAb monotherapy had a statistically significant reduction in tumor metastases in RF420 OS cells, with an even further reduction with the combination of hSFRP2 mAb and PD-1mAb. In contrast, in RF577 OS, metastases had a significant reduction with combination therapy but not with hSFRP2 mAb monotherapy. The average number of surface metastases in the RF577 model was almost 10 times lower than that of the RF420 model with IgG1 controls. As a consequence, it is possible that smaller degrees of variation in the number of metastases weighed more on the outcome in the RF577 model than in the RF420 model.

Serum SFRP2 has previously been shown to be a predictive biomarker for patients with breast cancer. Serum SFRP2 was found to be elevated in patients with breast cancer compared to a control group and was an independent prognostic predictor of progression-free survival [19]. In the present study, plasma SFRP2 was elevated in mice with OS lung metastases compared to tumor-free mice in two cell lines, and plasma SFRP2 was reduced in all treatment groups. At present we do not have an explanation for this intriguing finding. Given the importance of developing prognostic biomarkers to predict response to therapy, additional studies evaluating the potential of SFRP2 as a clinical prognostic or predictive biomarker of response in osteosarcoma patients are warranted.

## 5. Conclusions

Together with previously published data, this new study suggests that an anti-SFRP2 therapy may have broad applications in oncology and specifically, in metastatic osteosarcoma. Importantly, our results suggest that anti-SFRP2 therapy not only affects the tumor and endothelial compartments but also the tumor microenvironment through the calcineurin/NFATc3 signaling pathway, leading to a restoration of immunity. Finally, this new study shows that, through its ability to specifically downregulate CD38 and PD-1 expression, hSFRP2 mAb counteracts resistance to checkpoint inhibitors. It will be crucial in the future to expand upon this research and investigate the efficacy of hSFRP2 mAb on other cancers with primary checkpoint inhibitor resistance to explore the full potential of this new therapy. In the future, clinical trials will determine whether anti-SFRP2 therapy will be safe and efficacious for osteosarcoma in humans.

## 6. Patents

Discovery of Novel Targets for Angiogenesis Inhibition, Patent application no. 61/053,397. Inventors: Nancy Klauber-DeMore, Pharmaceutical Combination for the Treatment of Cancer, Provisional patent #112746-99263, Inventor Nancy Klauber-DeMore.

## Data Availability

The authors confirm that the data supporting the findings of this study are available within the article and its Supplementary Materials.

## Supplementary Materials

The following are available online at https://www.mdpi.com/article/10.3390/cancers13112696/s1, Figure S1. Establishment of microscopic lung metastases on histology. C57BL/6 mice were injected iv via tail vein with OS RF420 or RF577 cells. To determine when lung metastases were established, mice were euthanized, and lungs were resected, paraffin embedded, and H&E stained for microscopic analysis. (**A**) Histology confirmation of lung metastases in vivo in RF420 on day 7 and (**B**) on day 11 in RF577 metastatic OS models. Arrows point to metastases. Figure S2. hSFRP2 mAb antimetastatic effects are additive to mPD-1 mAb treatment in osteosarcoma in vivo. Lungs from RF420 OS-bearing mice treated with IgG1 control (4 mg/kg, i.v., weekly; *n* = 12), mPD-1 mAb (200 ug/100 µL i.p., q3 days, *n* = 10), hSFPR2 mAb (4 mg/kg i.v., q3 days, *n* = 12) or a combination of both treatments (*n* = 11) were resected after 3 weeks of treatment, paraffin embedded, sectioned and H&E stained. For each sample, lung metastases counts were performed manually at 20x by standard light microscopy on 3 independent slices separated from each other by an interval of 200 µm. Compared to IgG1 control treatment, there was no significant reduction in surface metastases with PD-1 mAb alone (*p* = 0.69). hSFRP2 mAb reduced surface metastases significantly compared to control (*p* = 0.0031). The combination therapy resulted in a significant reduction of surface nodules compared to hSFRP2 mAb treatment (** *p* < 0.0001). The full western blot figures were published in supplementary materials.
