# Supplementary material for: Overcoming PD-1 Inhibitor Resistance with a Monoclonal Antibody to Secreted Frizzled-Related Protein 2 in Metastatic Osteosarcoma
Source: Cancers (Basel). 2021 May 30;13(11):2696. doi: 10.3390/cancers13112696 (PMC8199140; doi:10.3390/cancers13112696)
Supplement: Supplementary file 1 [file cancers-13-02696-s001.zip › cancers-1218325-Figures S1 and S2.pdf]

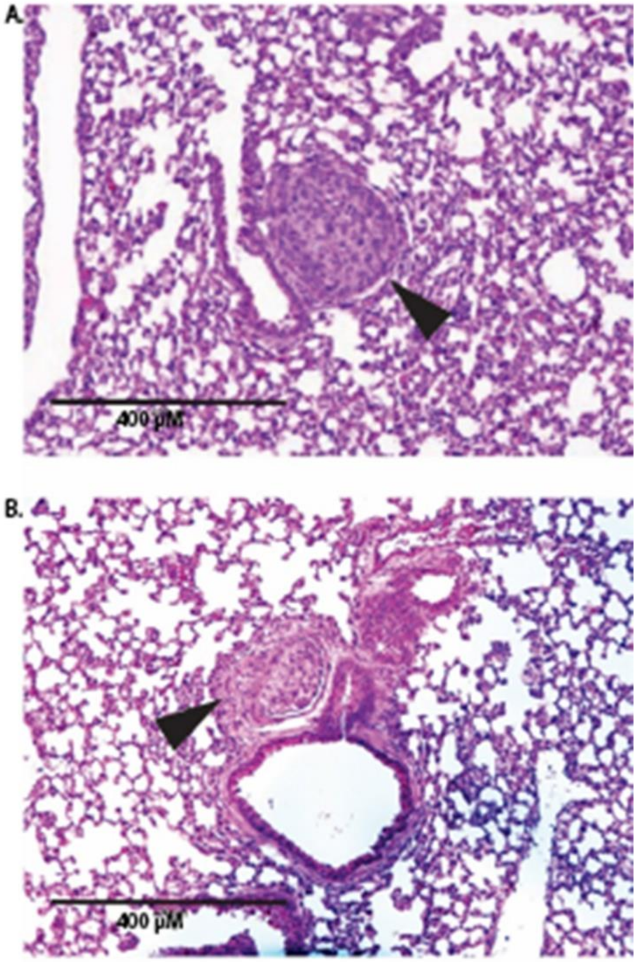

Figure S1. Establishment of microscopic lung metastases on histology. C57BL/6 mice were injected iv via the tail vein with OS RF420 or RF577 cells. To determine when lung metastases were established, mice were euthanized, and lungs were resected, paraffin embedded, and H&E stained for microscopic analysis. (A) Histology confirmation of lung metastases in vivo in RF420 on day 7 and (B) on day 11 in RF577 metastatic OS models. Arrows point to metastases.

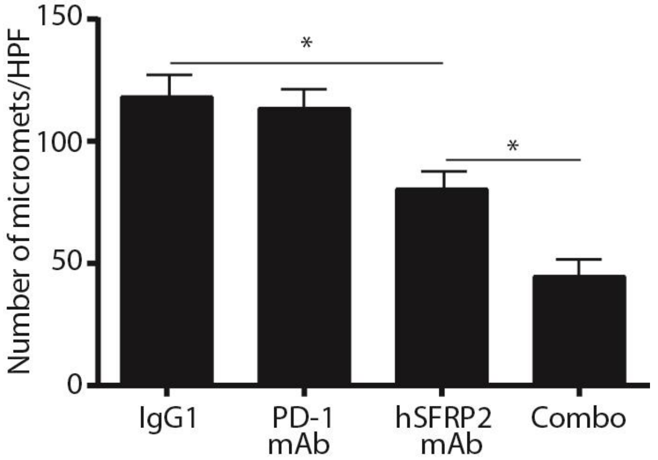

Figure S2. hSFRP2 mAb antimetastatic effects are additive to mPD-1 mAb treatment in osteosarcoma in vivo. Lungs from RF420 OS-bearing mice treated with IgG1 control (4 mg/kg, i.v., weekly; n = 12), mPD-1 mAb (200 µg/100 µL i.p., q 3 days, n = 10), hSFPR2 mAb (4 mg/kg i.v., q 3 days, n = 12) or a combination of both treatments (n = 11) were resected after 3 weeks of treatment, paraffin embedded, sectioned and H&E stained. For each sample, lung metastases counts were performed manually at 20X by standard light microscopy on 3 independent slices separated from each other by an interval of 200 µm. Compared to IgG1 control treatment, there was no significant reduction in surface metastases with PD-1 mAb alone ($p = 0.69$). hSFRP2 mAb reduced surface metastases significantly compared to control ($p = 0.0031$). The combination therapy resulted in a significant reduction of surface nodules compared to hSFRP2 mAb treatment (** $p < 0.0001$).
