# Supplementary figures and images for: Overcoming PD-1 Inhibitor Resistance with a Monoclonal Antibody to Secreted Frizzled-Related Protein 2 in Metastatic Osteosarcoma
Source: Cancers (Basel). 2021 May 30;13(11):2696. doi: 10.3390/cancers13112696 (PMC8199140; doi:10.3390/cancers13112696)

Fig. 1 A

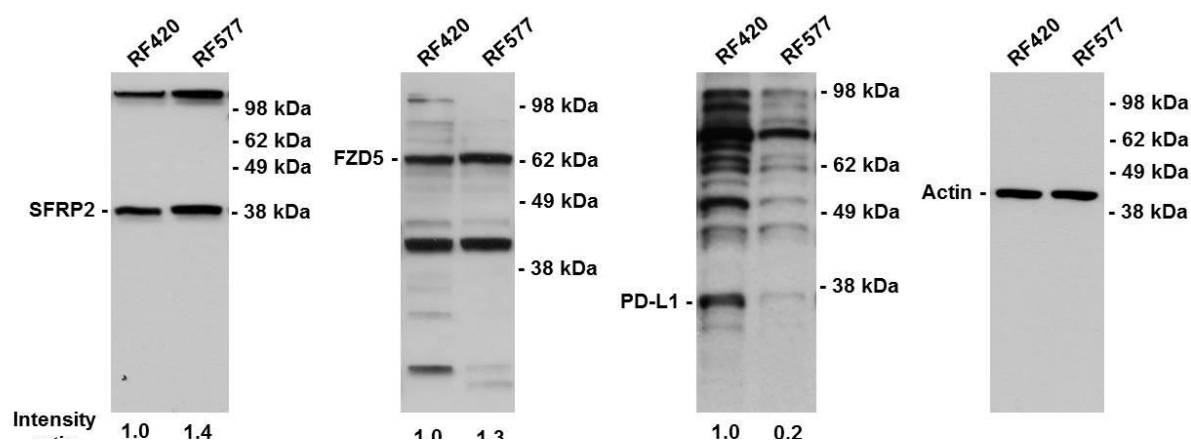

Fig. 1 A

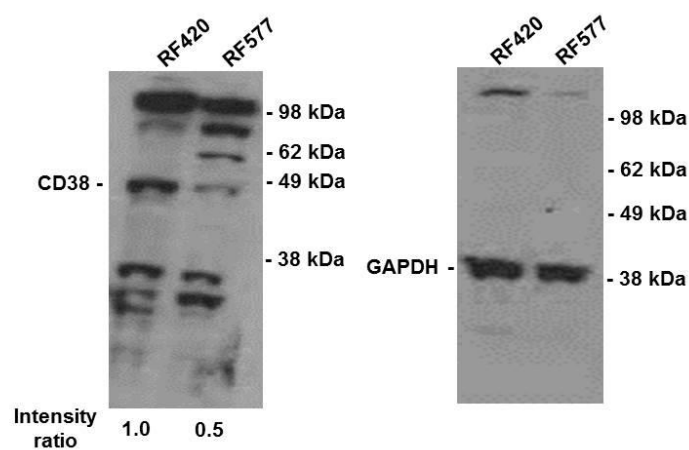

Fig. 5

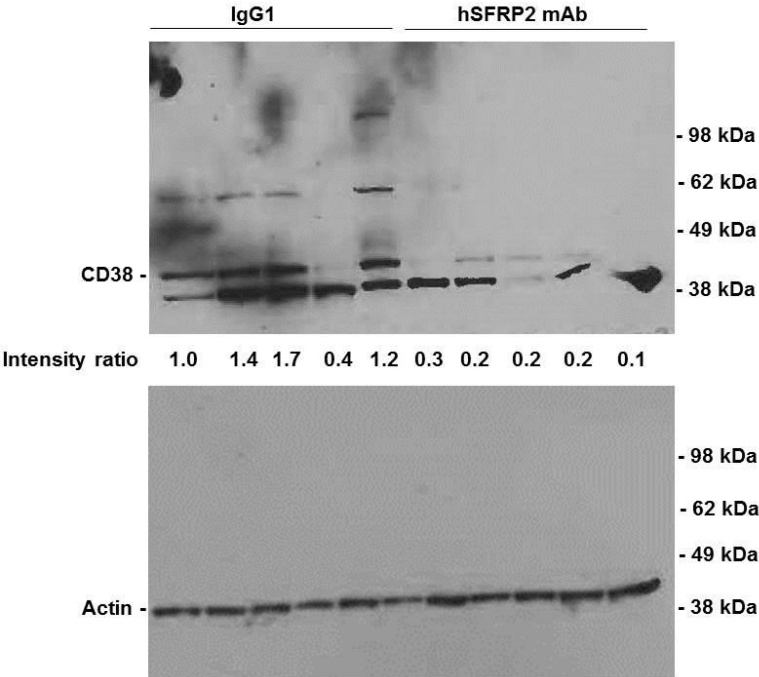

Fig. 6B

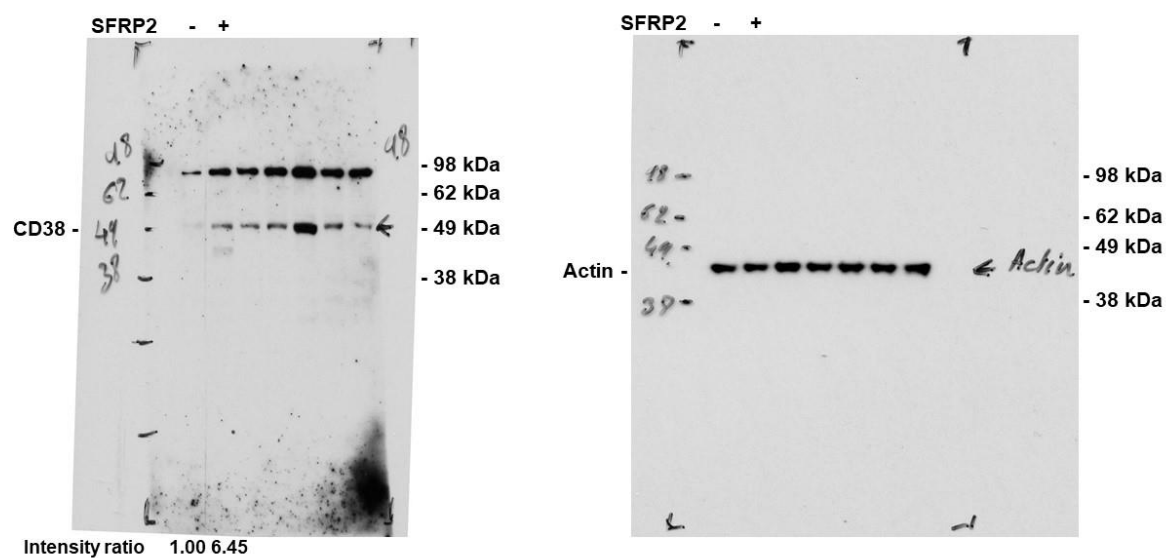

Fig. 6C

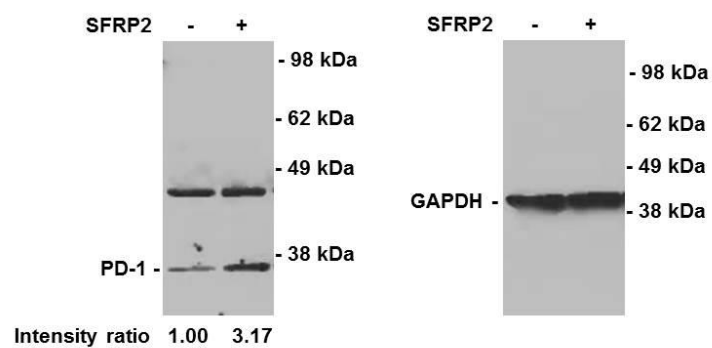

Fig. 6D

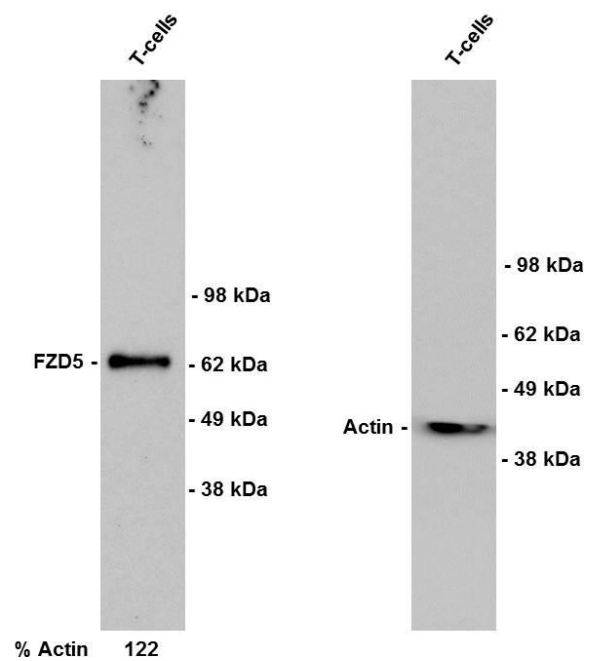

Fig. 6E

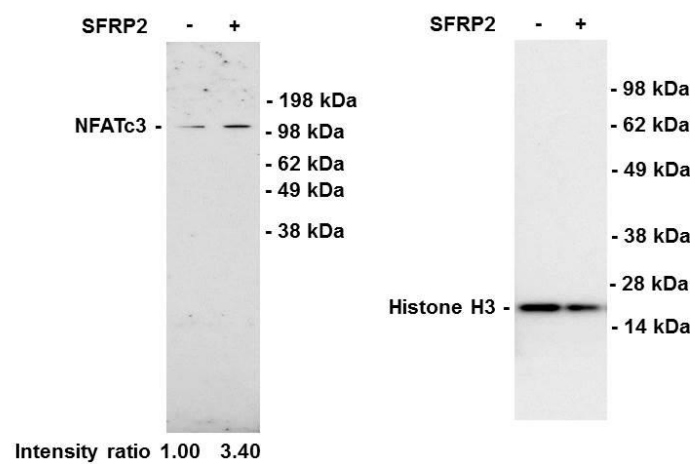

Fig. 6E

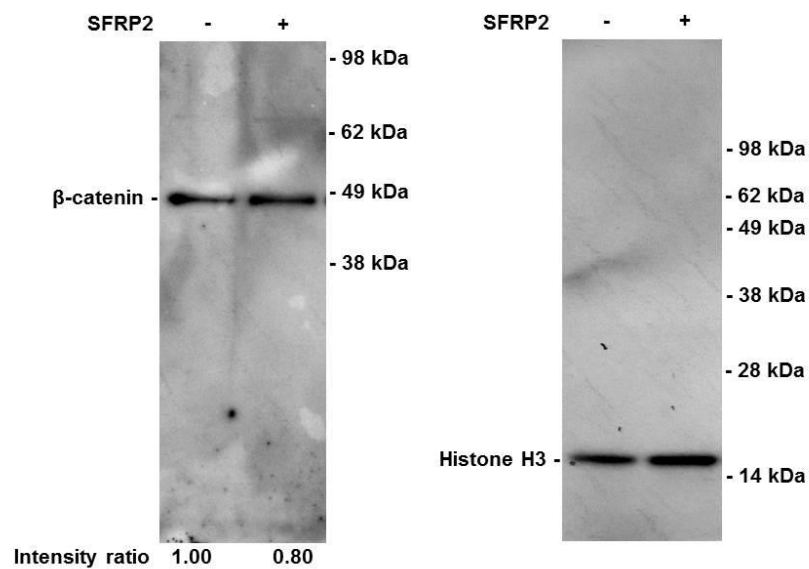

**Fig. 6F**

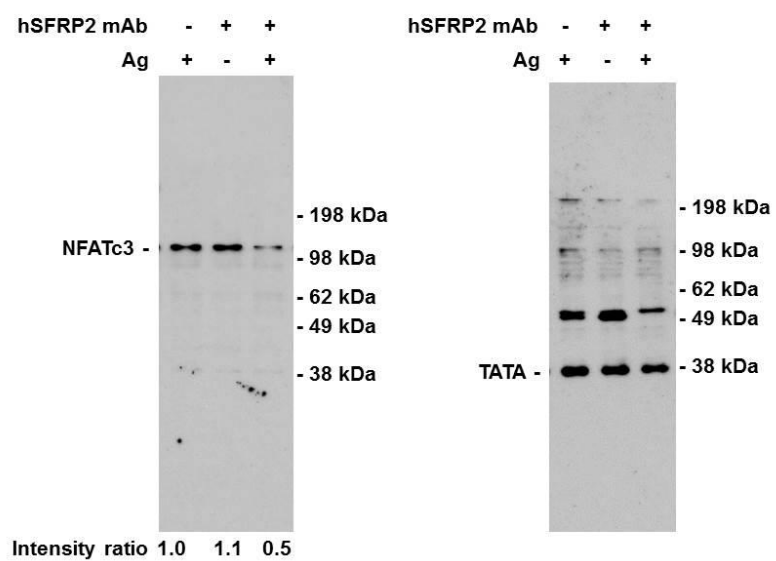

Fig. 7A

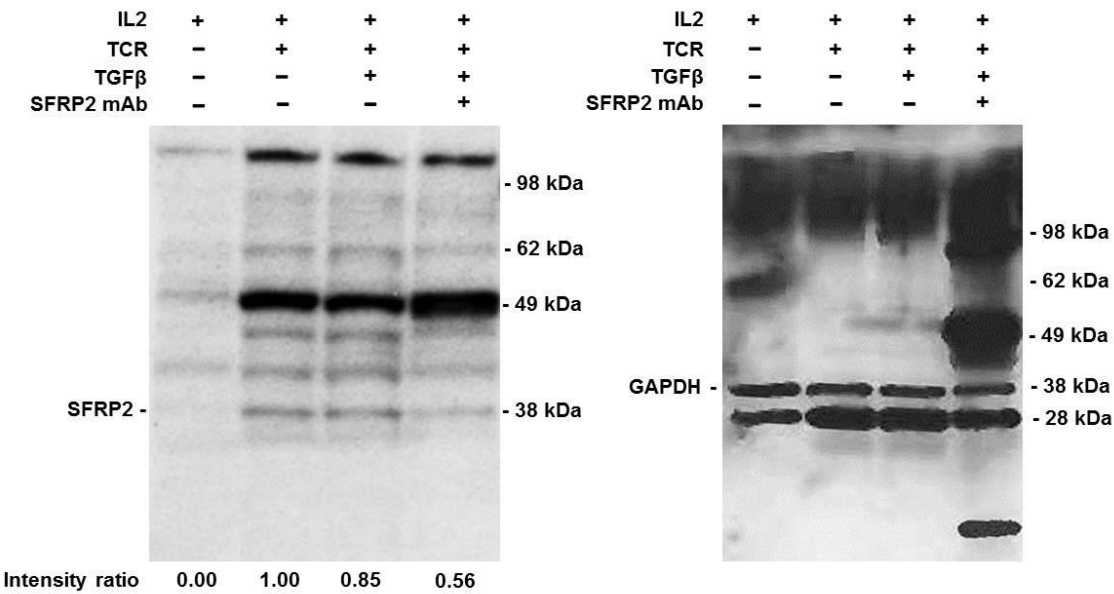

Fig. 7F

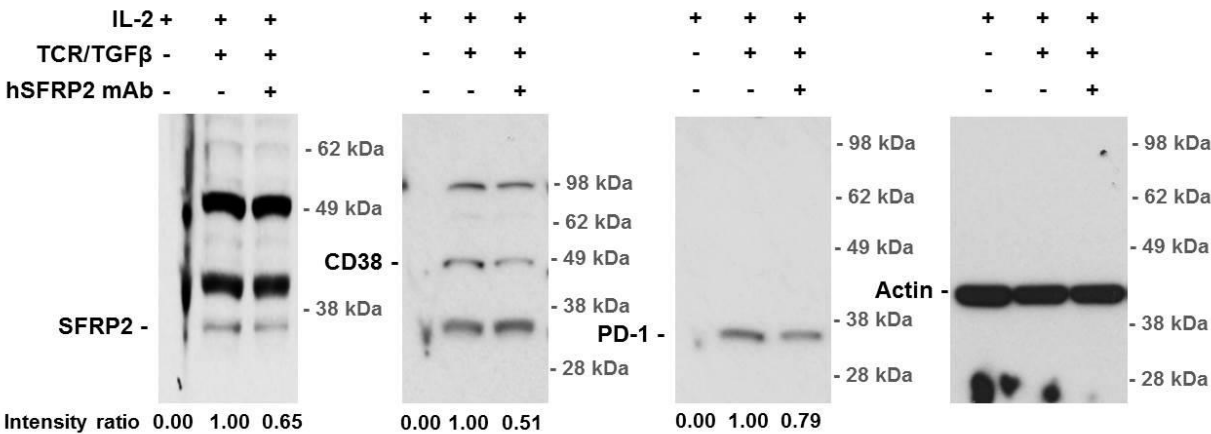

Supplement: Supplementary file 1 [file cancers-13-02696-s001.zip › cancers-1218325-WB figures.pdf]
